# Self‐optimizing Cobalt Tungsten Oxide Electrocatalysts toward Enhanced Oxygen Evolution in Alkaline Media

**DOI:** 10.1002/anie.202424074

**Published:** 2025-02-12

**Authors:** Christean Nickel, David Leander Troglauer, Zsolt Dallos, Dhouha Abid, Kevin Sowa, Magdalena Ola Cichocka, Ute Kolb, Boris Mashtakov, Bahareh Feizi Mohazzab, Shikang Han, Leon Prädel, Lijie Ci, Deping Li, Xiaohang Lin, Minghao Hua, Rongji Liu, Dandan Gao

**Affiliations:** ^1^ Department of Chemistry Johannes Gutenberg University Mainz Duesbergweg 10–14 Mainz 55128 Germany; ^2^ Research Division of Electron Crystallography Technical University of Darmstadt Schnittspahnstr. 9 64287 Darmstadt Germany; ^3^ Department for Molecular Spectroscopy Max Planck Institute for Polymer Research Ackermannweg 10 55128 Mainz Germany; ^4^ State Key Laboratory of Advanced Welding and Joining School of Materials Science and Engineering Harbin Institute of Technology (Shenzhen) Shenzhen 518055 People's Republic of China; ^5^ Key Laboratory for Liquid-Solid Structural Evolution and Processing of Materials Ministry of Education School of Materials Science and Engineering Shandong University Jinan 250061 People's Republic of China; ^6^ School of Energy and Power Engineering Shandong University Jinan 250061 People's Republic of China

**Keywords:** electrocatalysis, metal oxides, self-optimization, oxygen evolution reaction, active sites

## Abstract

Self‐optimizing mixed metal oxides represent a novel class of electrocatalysts for the advanced oxygen evolution reaction (OER). Here, we report self‐assembled cobalt tungsten oxide nanostructures on a lab‐synthesized copper oxide substrate through a single‐step deposition approach. The resulting composite exhibits remarkable self‐optimization behavior, shown by significantly reduced overpotentials and enhanced current densities, accompanied with substantial increase in OER kinetics, electrocatalytically active surface area, surface wettability, and electrical conductivity. Under operating conditions, interfacial restructuring of the electrocatalyst reveals the in situ formation of oxidized cobalt species as the true active site. Complementary density functional theory (DFT) calculations further demonstrate the formation of *OOH intermediate as the rate‐determining step of OER, and highlight the adaptive binding of oxygen intermediates, which transitions from tungsten to cobalt site during OER process. Our study provides a fundamental understanding of the self‐optimization mechanism and advances the knowledge‐driven design of efficient water‐splitting electrocatalysts.

## Graphical abstract

Progressively enhanced oxygen evolution is achieved by self‐optimization of cobalt tungsten oxide deposited on lab‐synthesized copper oxide substrate. Experimental data proved dynamic compositional / structural evolution while theoretical calculation revealed active site adaptation. The strategic design concept could strengthen the emergence of self‐improved electrocatalysts for a wide range of challenging energy conversion and storage reactions.

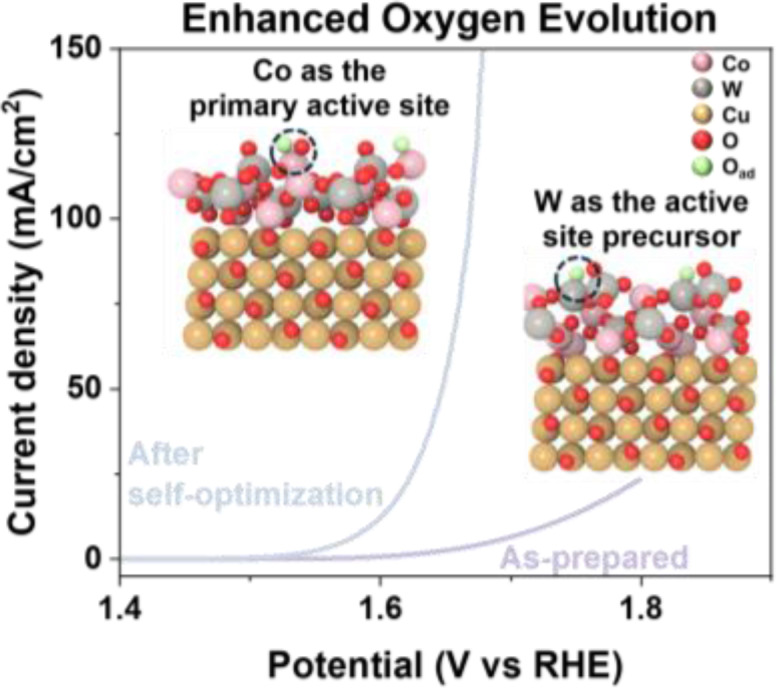

## Introduction

The electrochemical splitting of water into hydrogen and oxygen is a pivotal process for achieving carbon‐neutral energy production. When coupled with renewably “green” electricity sources, this approach can form the basis of a fully sustainable energy scheme.[^1^, ^2^] However, significant challenges remain, including the highly oxidative conditions, slow kinetics of proton‐coupled multielectron transfer, and substantial energetic activation barriers associated with the indispensable oxygen evolution reaction (OER).[^3^, ^4^, ^5^] Traditionally, noble metal oxides such as RuO_2_ ^[6]^ and IrO_2_,^[7]^ known for their high activity toward OER, have been the benchmark electrocatalysts. Nevertheless, the large‐scale use of these materials is severely hindered by their rarity, high cost, and susceptibility to degradation in both acidic and alkaline environments.^[8]^

In response to the demand for more sustainable electrocatalysts, research has increasingly focused on the development of industrially viable alternatives based on inexpensive earth‐abundant metal compounds. Notably, 3d to 5d transition metal oxides, particularly those incorporating Co, Ni, Fe, Cu and W,[^9^, ^10^, ^11^, ^12^, ^13^] have garnered significant attention due to their impressive catalytic activity and robust long‐term durability, especially when formulated as mixed metal (e. g., Ni−Co,[^14^, ^15^] Ni−Fe,^[15]^ Co−Fe,[^16^, ^17^] Cu−Co,[^18^, ^19^] Cu−W,^[20]^ Ni−W,[^21^, ^22^] Fe−W,^[23]^ Co−W,^[24]^ Fe‐Ni−W,^[25]^ Co−Ni‐Fe,^[26]^ Co‐Ni−W,^[27]^ Co−Fe−W,[^28^, ^29^] Co−Cu−W[^30^, ^31^]) oxides. In this regard, the underlying synergistic effects within these mixed metal oxides are critical for improving the OER performance grounded on enriched reaction active sites, boosted chemical / structural stability, or optimized electronic structures,[^32^, ^33^, ^34^, ^35^, ^36^] thereby enabling more efficient water splitting process.

In particular, self‐optimizing mixed metal oxides have been recognized as a novel class of OER electrocatalysts, characterized by progressively enhanced performance during operation. For example, Yamada et al. synthesized a tetravalent perovskite oxide, CaFe_0.5_Co_0.5_O_3_, demonstrating enhanced OER activity over 100 cyclic voltammetry (CV) tests attributed to the smaller charge‐transfer energies and formation of mixed Fe^4+^ / Co^4+^ active sites.^[37]^ In another example, Nguyen et al. developed a tungsten‐doped cobalt oxide film electrocatalyst that exhibited improved OER activity and kinetics, arising from enhanced surface wettability and increased electrochemically active surface area (ECSA) under O_2_‐evolving potentials.^[38]^ Recently, some of us have previously used molecular metal oxides (polyoxometalates, POMs) as well‐defined single‐source‐precursors for OER electrocatalysts, where cobalt‐functionalized polyoxotungstate ([Co^II^_4_(H_2_O)_2_(PW_9_O_34_)_2_]^10−^, Co_4_POM) was immobilized on commercial TiO_2_ using the cationic polymer polyethylenimine (PEI) as a linking agent.^[39]^ The resulting composite OER electrocatalyst demonstrated a unique self‐activation behavior, owing to the re‐structuring of Co_4_POM pre‐electrocatalyst. This led to the in situ formation of highly active Co^III^ oxide and / or hydroxide moieties, which was accompanied by an increase in both ECSA and electrical conductivity.

Despite their promotion of sustainable OER, the design of desired catalysts capable of enhanced OER electrocatalysis is still in its infancy. Moreover, the chemical adaptations during the self‐optimization involving surface re‐structuring, generation of new phases and alteration of metal oxidation state are largely unknown.^[40]^ To address these critical challenges, advances in experimental and computational electrochemical surface analyses are urgently required to reveal the self‐optimization mechanism and therefore drive the knowledge‐based design of next‐generation self‐optimizing OER electrocatalysts.[^41^, ^42^] Furthermore, the development of viable and scalable deposition approaches is of utmost technological, economic and ecological significance, enabling stable anchoring of OER pre‐catalysts on selected promising substrates with high mechanical integrity.^[43]^

In this work, we report a facile one‐step robust deposition of self‐assembled mixed Co−W oxides on lab‐synthesized CuO (L−CuO) microflower substrate using varying Co^2+^ / [SiW_11_O_39_]^8−^ precursor molar ratios of 1 : 1 (composite **1**), 2 : 1 (composite **2**), 3 : 1 (composite **3**) and 4 : 1 (composite **4**), respectively. When operated in 1.0 M aqueous KOH, the resulting **3** exhibits a most remarkably self‐optimizing catalytic performance with progressively decreased overpotentials and Tafel slopes, as well as increased peak current densities, ECSA, surface wettability and electrical conductivity. Explicitly, post‐catalytic X‐ray photoelectron spectroscopy (XPS) proved the oxidized valence state of the Co and W metal center. Furthermore, OER process‐dependent attenuated total reflection Fourier transform infrared (ATR‐FTIR) spectroscopy and Raman spectroscopy measurements revealed the dynamic interfacial processes / surface re‐structuring that governs boosted catalytic performance at the molecular level. At the atomic level, density functional theory (DFT) calculations revealed key insights into OER self‐optimization mechanism. The formation of the *OOH intermediate was identified as the rate‐determining step of the reaction. Additionally, the DFT calculations highlighted the dynamic transformation of active sites during the OER process, alongside a notable reduction in overpotential and enhanced electrical conductivity following the self‐optimization of **3**.

This initial proof of concept paves the way for the development of self‐assembled proficient electrocatalysts, in which molecular precursors are transformed into self‐optimizing nanostructures to promote multi‐electron electrocatalysis.

## Results and Discussion

### Electrocatalyst Fabrication and Characterization

**Figure 1:**
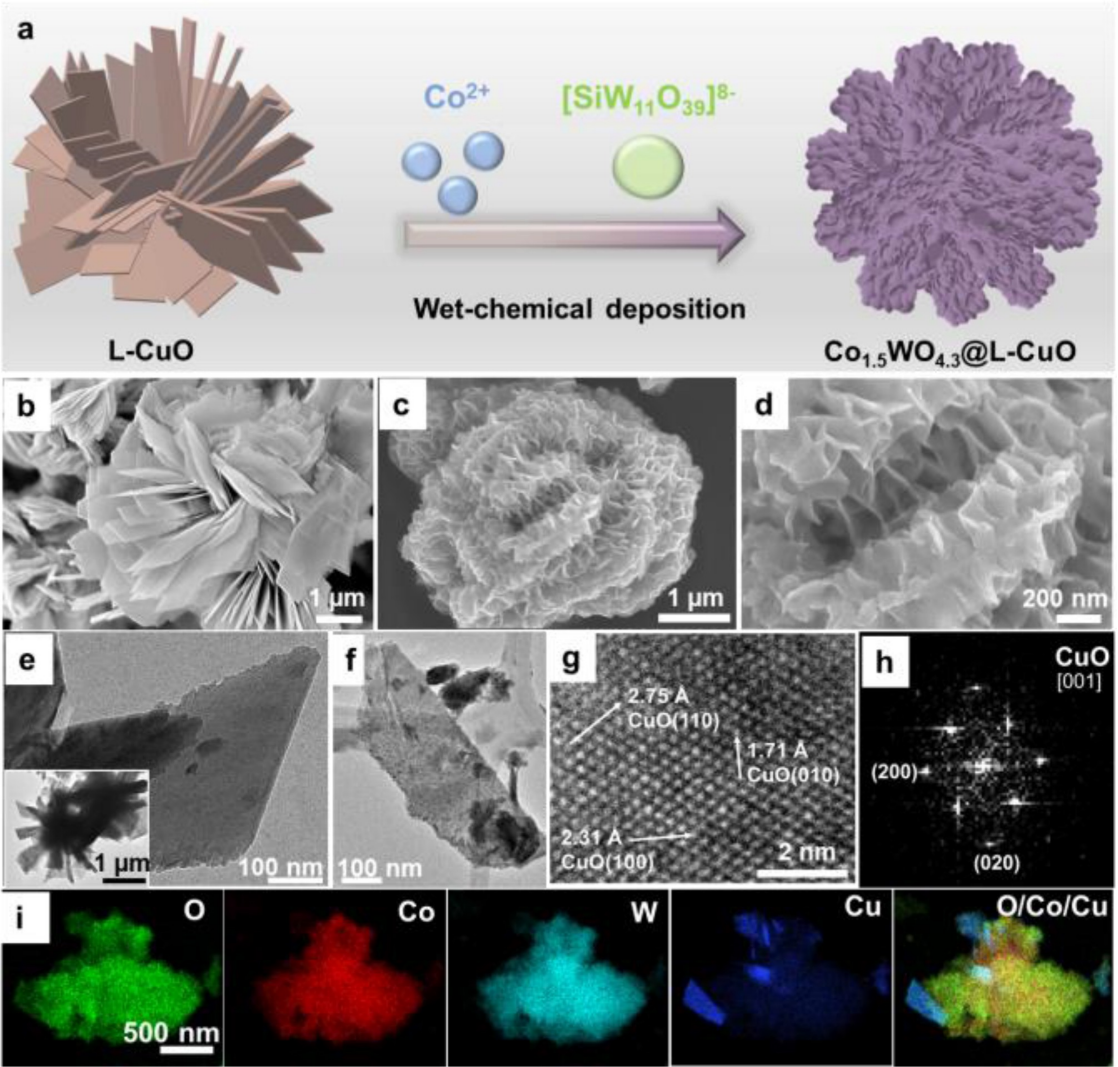
Synthetic illustration, structural and elemental analysis of **3**. (a) Schematic materials design and fabrication approach leading to mixed metal oxide composite. (b) SEM image of L−CuO, (c–d) SEM images of **3**. (e) TEM image of L−CuO nanopetal and microflower (inset). (f) TEM image of **3**. (g) HRTEM and (h) fast Fourier‐transformation (FFT) of **3**, showing a matching reflection of CuO crystalline substrate. (i) STEM‐EDX elemental mappings, showing the elemental distribution of O, Co, W and Cu (for HAADF‐STEM image of the particle, see the SI, **Figure S8**).

**Figure 2:**
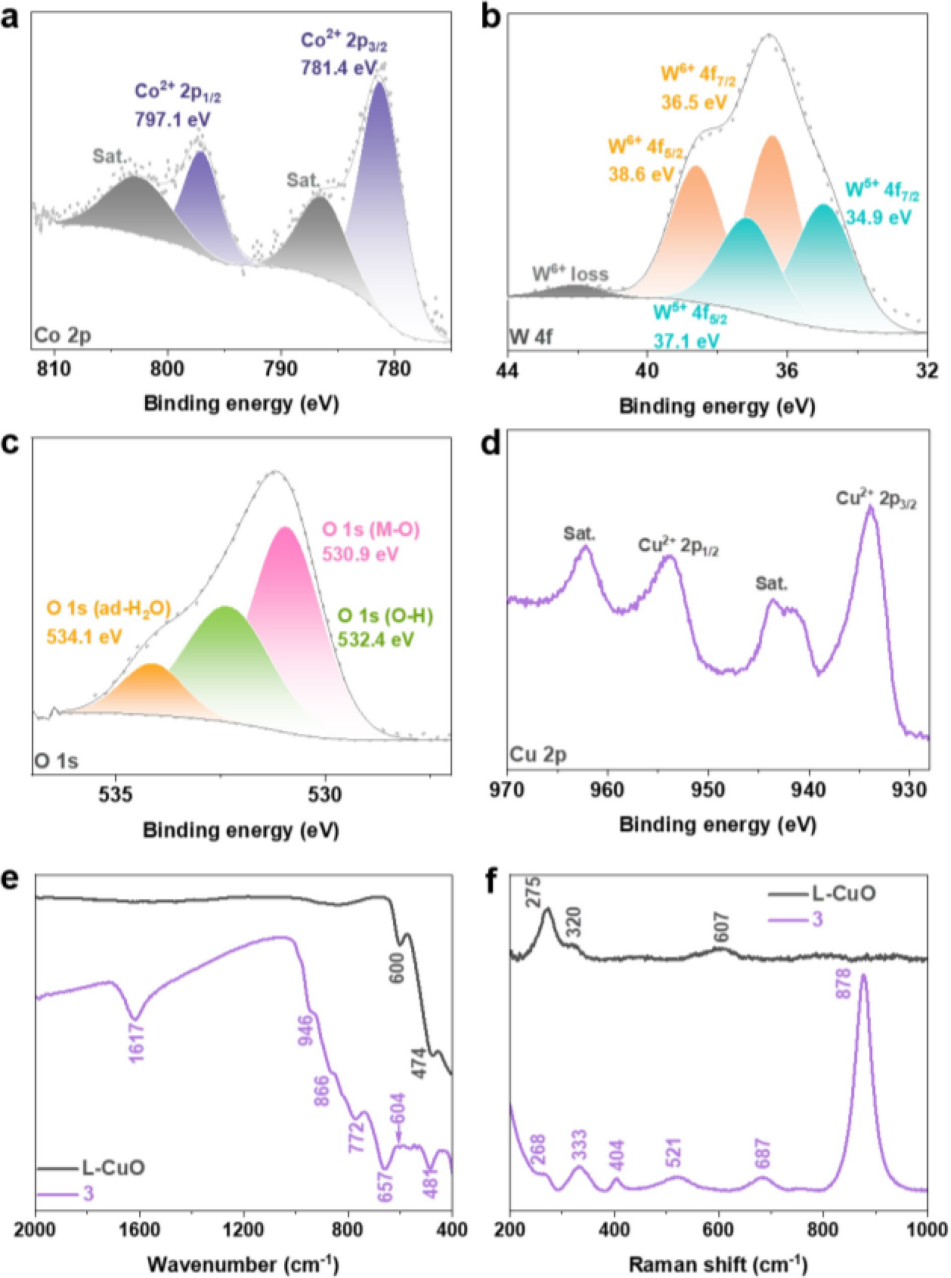
XPS spectra for (a) Co 2p, (b) W 4 f, (c) O 1s and (d) Cu 2p of **3**. (e) ATR‐FTIR spectra and (f) Raman spectra of **3** and L−CuO.

Here, we present a scalable fabrication method for mixed metal oxides through a simple one‐pot wet‐chemical deposition process (Figure 1a). This approach yields a highly nanostructured composite, which serves as a promising self‐optimizing electrocatalyst for enhanced alkaline OER. The electrocatalyst design was grounded in the L−CuO documented in our previous work.^[44]^ Corresponding preparation procedures are available in the Supporting Information (SI). As illustrated in scanning electron microscopy (SEM, Figures 1b and **S1**) and transmission electron microscopy (TEM, Figure 1e and inset, **Figure S2**), the L−CuO features a flower structure with a diameter of 3–5 μm. Energy‐dispersive X‐ray (EDX) elemental mapping showed a uniform distribution of the constituent Cu and O elements (**Figure S3**). Powder X‐ray diffraction (pXRD, **Figure S4**) revealed the presence of characteristic crystalline CuO, which is a known semiconductor with high electron mobility^[45]^ and has recently been employed for OER electrocatalysis.[^44^, ^46^, ^47^] In addition, ATR‐FTIR spectroscopy and Raman spectroscopy further identified characteristic Cu−O vibrational modes (Figures 2e–f). Subsequent modification of the L−CuO was achieved by one‐step hydrothermal deposition using molecular precursors (Co(NO_3_)_2_ ⋅ 6H_2_O and K_8_[SiW_11_O_39_] ⋅ 13H_2_O, Co^2+^ / [SiW_11_O_39_]^8−^ molar ratio of 3 : 1) at 180 °C for 8 h, leading to the self‐assembled Co−W oxide nanostructures rooted on L−CuO substrate (composite **3**) as demonstrated by SEM (Figures 1c–d and **S5**) and TEM (Figure 1f). High‐resolution TEM (HRTEM, Figure 1g) and fast Fourier‐transformation analysis of the same area (Figure 1h) reveal that **3** is based on CuO crystalline substrate. This observation indicates the amorphous nature of deposited mixed Co−W oxide (**Figure S6**), which is in accord with the pXRD pattern (**Figure S7a**). In addition, EDX elemental mappings from scanning TEM (STEM) indicate that the deposited catalyst contains O, Co and W, while the presence of Cu nanopetal is assigned to signals arising from the L−CuO substrate (Figures 1i and **S8**).

Further, XPS was employed to investigate more detailed chemical structures and the surface oxidation states of **3**. The XPS survey spectrum verifies the presence of Co, W, Cu and O (**Figure S9**). In detail, the Co 2p region reveals the presence of Co^2+^ (Figure 2a), while W 4 f region indicates the coexistence of W^5+^ and W^6+^ in a ratio of approximately 1 : 1.3 (Figure 2b).^[48]^ The O 1s region displays three distinct features corresponding to metal‐oxygen bonds (M−O, including Co−O, W−O, and Cu−O), hydroxyl groups (O−H), and physically adsorbed water (ad‐H_2_O), respectively (Figure 2c).[^30^, ^49^] Additionally, the Cu 2p region confirms the presence of Cu^2+^ originating from the L–CuO substrate (Figure 2d).^[50]^ The presence of the mixed metal oxide was further corroborated by ATR‐FTIR (Figure 2e) and Raman (Figure 2f) spectroscopy. Specifically, the ATR‐FTIR spectrum revealed a peak at 946 cm^−1^, corresponding to the W=O bond, while the peaks at 866 cm^−1^ and 772 cm^−1^ were attributed to W−O−W bonding.[^51^, ^52^] Additionally, the Co−O vibrational mode was detected at 657 cm^−1^.^[53]^ The characteristic bands associated with Cu−O were identified at 604 cm^−1^ and 481 cm^−1^,^[30]^ which exhibited a blue shift compared to those of the L−CuO (600 cm^−1^ and 474 cm^−1^), suggesting an interaction between Co−W oxide and L−CuO substrate.^[54]^ Complementarily, the characteristic vibrational modes of **3** were also observed in the Raman spectra (W=O: 878 cm^−1^, W−O−W: 687 cm^−1^, 333 cm^−1^,^[55]^ Co−O: 521 cm^−1^, 404 cm^−1^,^[30]^ Cu−O: 268 cm^−1[56]^). In addition, ATR‐FTIR spectroscopy identified O−H vibrations and thermogravimetric analysis (TGA) indicated approximately 6.7 wt‐% water of hydration (**Figure S10**). As measured by inductively coupled plasma optical emission spectroscopy (ICP‐OES), the atomic ratio of Co : W in **3** is determined to be 1.5 : 1 (**Figure S11**). In summation, the composite **3** synthesized is best described as a mixed Co−W oxide deposited on L−CuO with an approximate formula of Co_1.5_WO_4.3_@L−CuO ⋅ xH_2_O.

To assess the role of the Co / W precursors in the synthesis, we performed identical syntheses in varying Co^2+^ / [SiW_11_O_39_]^8−^ molar ratios on L−CuO substrate (**Table S1**), namely, 1 : 1 (composite **1**), 2 : 1 (composite **2**), 4 : 1 (composite **4**), as well as in the presence of only Co^2+^ (composite **5**) and [SiW_11_O_39_]^8−^ (composite **6**). Additionally, to elucidate the role of CuO support, commercial CuO (C−CuO) was employed for synthesis in Co^2+^ / [SiW_11_O_39_]^8−^ molar ratio of 3 : 1, resulting in composite **7** (**Table S1**). All the resulting composites were used as references to compare the electrocatalytic performance of **3**.

Based on the ICP‐OES result (**Figure S11**), the atomic ratios of Co / W in **1**, **2, 4** and **7** were revealed to be 0.1 : 1, 0.3 : 1, 1.9 : 1 and 0.2 : 1, respectively. ATR‐FTIR data indicate that, in addition to the characteristic Cu−O vibration modes originating from the L−CuO or C−CuO substrate, the co‐existence of W−O and Co−O vibration modes was observed for **2**, **3**, **4** and **7** (while it is rather less observable for **1**, **Figures S12a**, **c**). As expected, **5** and **6** feature Co−O and W−O vibrational modes, respectively (**Figure S12b**). Furthermore, pXRD data demonstrate that **1** and **6** retain the crystallography of L−CuO substrate, while **2**–**4** show reduced crystallinity, and composite **5** is based on newly formed Cu−Co oxide crystalline on the L−CuO substrate (**Figure S7a**). Notably, no considerable change in crystallography is observed for **7** as compared to the C−CuO substrate (**Figure S7b**), indicative of the amorphous structure of deposited Co−W oxide catalyst. Furthermore, the oxidation states of the metal centers are revealed by XPS analysis, verifying the presence of Co^2+^ and W^6+^ (**Figure S13**). Together with the ICP data (**Figure S11**), the composite **7** can be indexed as Co_0.2_WO_3.2_@C−CuO.

Moreover, both the Brunauer–Emmett–Teller (BET) specific surface area and the Barrett–Joyner–Halenda (BJH) pore size distribution (**Figures S14**–**S16** and **Table S2**) of **3** showed a considerable increase (18.38 m^2^/g and 16.32 nm) compared to the L−CuO substrate (11.16 m^2^/g and 11.42 nm). These enhancements are conducive to improving electrocatalytic OER activity, owing to the increased exposure of active sites and enhanced mass transfer of electrolytes.^[18]^ Interestingly, **1** and **2** show relatively lower surface areas in comparison to **3**, while **4–6** display unexpectedly lower values than L−CuO substrate. Note that, **7** exihbits higher surface area (26.505 m^2^/g) while lower pore size (12.232 nm) than **3**. Moreover, the pore size distribution features dynamic variation. Supplementarily, SEM data of **1**, **2**, **4**, **5** and **6** demonstrate differrent morphologies compared to **3** (**Figure S17**), while surface aggregation was observed specifically for **5**, showing the lowest surface area (**Table S2**). Furthermore, with C−CuO as the substrate which features micro‐scale particles and much lower surface area compared to L−CuO (**Figure S18a**, **Table S2**), **7** represents unassociated surface mophology to **3** (**Figure S18b**), indicative of the crucial role of CuO microstructure for the deposition of self‐assembled Co−W oxide nanstructures.

### Electrocatalytic OER Performance

**Figure 3:**
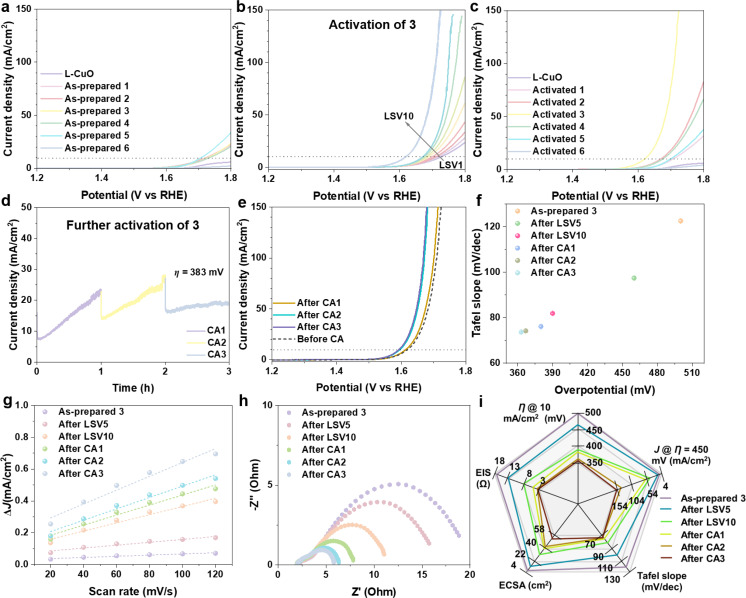
Electrocatalytic OER performance of **1**–**6**, and L−CuO in 1.0 M aqueous KOH. (a) Initial LSV curves of as‐prepared **1**–**6** and L−CuO (first scan, LSV1). (b) Activation process of **3** by ten successive LSV scans (LSV1–LSV10). (c) LSV curves of activated **1**–**6** (after respective successive LSV scans) and L−CuO. (d) Stepped chronoamperometry (CA) test of **3** over a period of 3 h. (e) LSV curves of activated **3** before and after CA tests. (f) Tafel slopes and overpotentials of **3** during the studied OER process. (g) ECSA study of **3**, showing the corresponding linear fits. (h) Electrochemical impedance spectroscopy (Nyquist plots) of **3** measured at the overpotential *η*=400 mV. (i) Summary of performance metrics for **3** during the studied OER process.

In the next step, we assessed the electrocatalytic OER performance of the mixed metal oxide composites under alkaline media (1.0 M aqueous KOH, pH 13.8, room temperature) using a H‐cell with standard three‐electrode configuration. All potentials were converted to the reversible hydrogen electrode (RHE) as a reference. The electrocatalytic activity of the as‐prepared **1**–**6** and L−CuO was initially evaluated using linear sweep voltammetry (LSV). As illustrated in Figure 3a, the L−CuO exhibits minimal OER activity. In contrast, **1**–**4** demonstrate comparable behaviors, characterized by increased but still relatively limited OER activity, as well as similar Tafel slopes (**Figure S19**). Notably, **5** shows the highest OER activity and the lowest Tafel slope attributed to the formation of Cu−Co oxide crystalline on CuO substrate, whereas **6** displays the lowest OER activity with a similar Tafel slope to **1**–**4** (**Figure S19**).

Subsequently, the self‐optimization of **1**–**6** toward enhanced OER activity was observed by successive LSV scans (Figures 3b and **S20a**–**e**). Specifically, **1** and **5** displayed finite self‐optimization behavior, reaching stabilized OER activity after LSV2 and LSV8, respectively. In contrast, **2**, **3**, **4** and **6** demonstrate a more sustained increase in OER activity over 10 LSV scans. Note that, the self‐optimization procedure is absent for the L−CuO (**Figure S20f**), underscoring the critical role of the deposited mixed Co−W oxide in enhancing OER performance. The LSV curves for activated **1**–**6** and L−CuO are shown in Figure 3c. Remarkably, activated **3** exhibited the lowest onset potential, highest current density and lowest Tafel slope (**Figure S21a**), attributable to the optimal Co^2+^ / [SiW_11_O_39_]^8−^ molar ratio of the precursor solution (**Table S1**). Note that, activated **1**–**4** (Co−W oxide involving), and **6** (W oxide involving) displayed considerably decreased Tafel slopes, namely, enhanced OER kinetics while activated **5** (Co oxide involving, W oxide free) showed a marginal decrease in Tafel slope (**Figure S21b**). This suggests the pivotal role of the W oxides in improving OER kinetics during the successive LSV scanning process.

Further self‐optimization of **3** was next investigated and verified by the stepped chronoamperometry (CA, at an overpotential *η*=383 mV) over a period of 3 h. As displayed in Figure 3d, a considerable increase in current density was achieved for CA1 (from 7.5 mA/cm^2^ to 23.2 mA/cm^2^) and CA2 (from 14.0 mA/cm^2^ to 27.6 mA/cm^2^), while almost stable performance was observed for CA3 (from 16.5 mA/cm^2^ to 18.5 mA/cm^2^). Correspondingly, the LSV curves (Figure 3e) after CA1 and CA2 demonstrate a further enhancement in OER activity compared to the pre‐CA measurements, while maximized OER activity was achieved after CA3, featuring the lowest overpotential *η*=363 mV (at *J*=10 mA/cm^2^) and highest current density of 149.6 mA/cm^2^ (at *η*=450 mV). In this process, further reduced Tafel slopes (indicative of increased OER kinetics) were also obtained (Figure 3f and **Figure S22**). The self‐optimization process of **3** is summarized in Figure 3i and **Table S3**. More intriguingly, the maximum OER activity was maintained over a course of 15 h (**Figure S23**), despite a noticeable drop in current density during long‐term CA, due to the aggregation of O_2_ bubbles on the electrode surface that blocked the active sites. For comparison, CA measurement (at *η*=570 mV, <3 h, **Figure S24a**) was performed for the L−CuO substrate, which however shows degraded OER activity (**Figure S24b**).

In terms of the reference composite **7** (with self‐optimization free C−CuO as the substrate, **Figure S25a**), an unsatisfying self‐optimization behavior was observed compared to **3** (with self‐optimization free L−CuO as the substrate, **Figure S20f**). Briefly, it shows very marginal activation during successive LSV scans while considerable activation can be obtained after stepped CA tests (**Figures S25b**–**d**). Interestingly, the as‐prepared **7** demonstrates higher activity than that of as‐prepared **3**. Nevertheless, the activated **3** after CA3 substantially surpasses the activated **7** after CA3 (**Figure S25e**). This highlights the superiority of L−CuO as a desirable support for the deposition of self‐assembled Co−W oxides driving a significant self‐optimization process. Notably, when commercial Co oxide and W oxide are physically mixed with the L−CuO, no self‐optimization can be yielded at the same operation condition (**Figure S25f**), further indicating the indispensable self‐optimization behavior of the deposited Co−W oxides in **3**.

### Electrochemical Mechanistic Studies

To gain mechanistic insights underlying the unique self‐optimization OER process of **3**, we conducted further electrochemical analyses to determine the ECSA and the charge‐transfer resistance (*R*_ct_) at the electrode/electrolyte interface. Specifically, we analyzed the ECSA based on electrochemical double‐layer capacitance (*C*_dl_) using scan‐rate‐dependent CV (for details, see **Figure S26**). As shown in Figures 3g, **3 i**, **S27a** and **Table S3**, continuously increasing ECSA (from 4.6±0.4 cm^2^ to 54.4±0.4 cm^2^) is observed, representing nearly a twelve‐fold enhancement after CA3. This significant increase in surface area is attributed to the vigorous formation of O_2_ bubbles and the evolution of the electrocatalyst interface, potentially exposing a larger number of accessible active sites contributing to the OER self‐optimization process. Complementarily, analysis of the hydrophilicity of the electrode (**3** coated carbon paper) surface by the sessile drop method (1.0 M KOH as the medium) showed a gradual decrease in water contact angle (from 122.0° to 64.9°, **Figure S28**), indicative of increased surface wettability and therefore more sufficient electrolyte/catalyst interface contact. Furthermore, a gradient decrease in *R*_ct_ (from 17.0 Ω to 3.5 Ω) was revealed by electrochemical impedance spectroscopy (EIS). This suggests that the maximized optimization after CA3, characterized by the lowest *R*_ct_, enables the most efficient interfacial electron transfer (Figures 3h, **3i**, **S27b**, and **Table S3**). In comparison to **3**, the L−CuO substrate demonstrates substantially lower ECSA (2.7±0.1 cm^2^, **Figure S29**) and markedly higher *R*_ct_ (191.8 Ω, **Figure S30**). Moreover, the corresponding studies on as‐prepared **7** demonstrate higher ECSA and conductivity compared to those of as‐prepared **3**, while lower metrics were observed after CA3 (**Figure S31**), correlating to the OER activity comparison afore‐observed (**Figure S25e**).

Overall, **3** exhibits competitive performance in comparison to literature‐reported Co−W oxide‐based OER electrocatalysts (see **Table S4**), demonstrating comparable or superior metrics after undergoing a unique self‐optimization process.

### Electrocatalyst Interfaces Studying

To elucidate the origins of the self‐optimizing behavior of **3**, various spectroscopic approaches were used to investigate the electrocatalyst interfaces during the studied OER process. The post‐catalytic XPS analyses uncovered the underlying changes in the chemical composition and oxidation states of the elements. Throughout the studied OER process, the formation of higher oxidized metal species was observed, including the emergence of Co^3+[57]^ with an increased ratio to Co^2+^ (**Figure S32** and **Table S5**), as well as a rising proportion of W^6+^ compared to W^5+^ (**Figure S33** and **Table S5**). Notably, after LSV5 and LSV10, the metal‐oxygen (M−O) bonds and hydroxyl groups (O−H) remained present in composite **3**, while oxygen atoms in the sulfonic acid group of Nafion were also detected (**Figure S34**).^[58]^ As the OER process progressed (CA1–CA3), an increasing proportion of metal‐hydroxide (M‐OH) and oxyhydroxide (M‐OOH) species[^59^, ^60^] to metal‐oxygen (M−O) species (SI, **Table S5**) were observed.

In addition, the OER‐process‐dependent ATR‐FTIR spectra of **3** (after LSV5) show the appearance of a new signal at ~2980 cm^−1^ assigned to H‐bonded hydroxyl groups (O−H),^[61]^ which was intensified over the subsequent OER processes (LSV10–CA3). Notably, stretching vibration of the M‐OOH was observed after CA1, evidenced by an increasing peak at ~1020 cm^−1^,[^62^, ^63^] indicating its critical role as an active substance responsible for the progressively enhanced OER electrocatalysis.[^64^, ^65^] Complementing these findings, the Raman peaks ascribed to Co−O characteristic at 404 cm^−1[30]^ gradually diminished (as‐prepared – LSV10). Instead, the new peaks at 456 and 508 cm^−1^ became increasingly pronounced as the OER process progressed stepwise (CA1–CA3), which are associated with the stretching vibrations of Co‐OOH.[^66^, ^67^, ^68^] In addition, the W−O−W vibrational mode at 687 cm^−1^ underwent a red shift to a lower stretching frequency at 663 cm^−1^ after LSV5, attributed to the oxidized W valence state (SI, **Table S4**), and resulting modified electronic structure and W−O interaction strength.^[69]^ Based on these findings, we propose that the remarkable self‐optimization behavior is likely attributable to the in situ formation of OER‐active Co‐OOH species on the CuO substrate during OER electrocatalysis in alkaline media, accompanied by modifications in the electronic structure of W oxide with higher oxidation state.

To assess the potential decomposition of **3** and leakage of its components, ICP‐OES analysis was conducted on the electrolyte throughout the stepped CA tests. The analysis revealed that the concentrations of constituent metals (Co, W, and Cu) were below detectable limits, indicative of no metal leakage. These results underscore the exceptional mechanical stability of **3** during the progressively self‐optimizing process, despite significant chemical adaptations occurring at the electrocatalyst interface under harsh oxidative and alkaline conditions.

### Theoretical Calculations

In addition to the aforementioned insights at the molecular level, DFT calculations were performed to shed light on the catalytic mechanism of **3** at the atomic level. Building on the experimental XRD, TEM, XPS and ICP‐OES data, two amorphous surface models, namely, as‐prepared **3** and activated **3** after CA3 (**Figure S35**), were established based on ab initio molecular dynamics (AIMD) calculation and the melt‐quenched method (for details, see Section 2 in the SI). Notably, the surface model of activated **3** after CA3 incorporates additional oxygen atoms to replicate the chemical adaptation, e. g., in oxidation states and elemental ratios.

**Figure 4:**
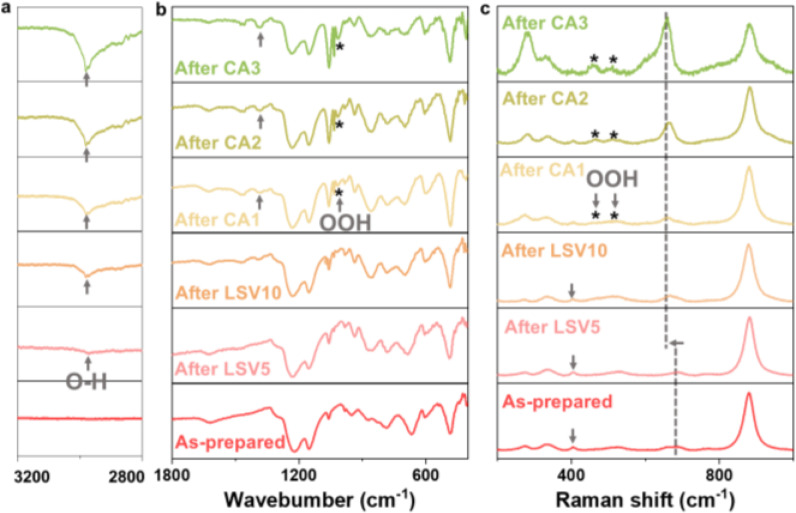
OER process‐dependent ATR‐FTIR (a, b) and Raman (c) spectra of **3**.

**Figure 5:**
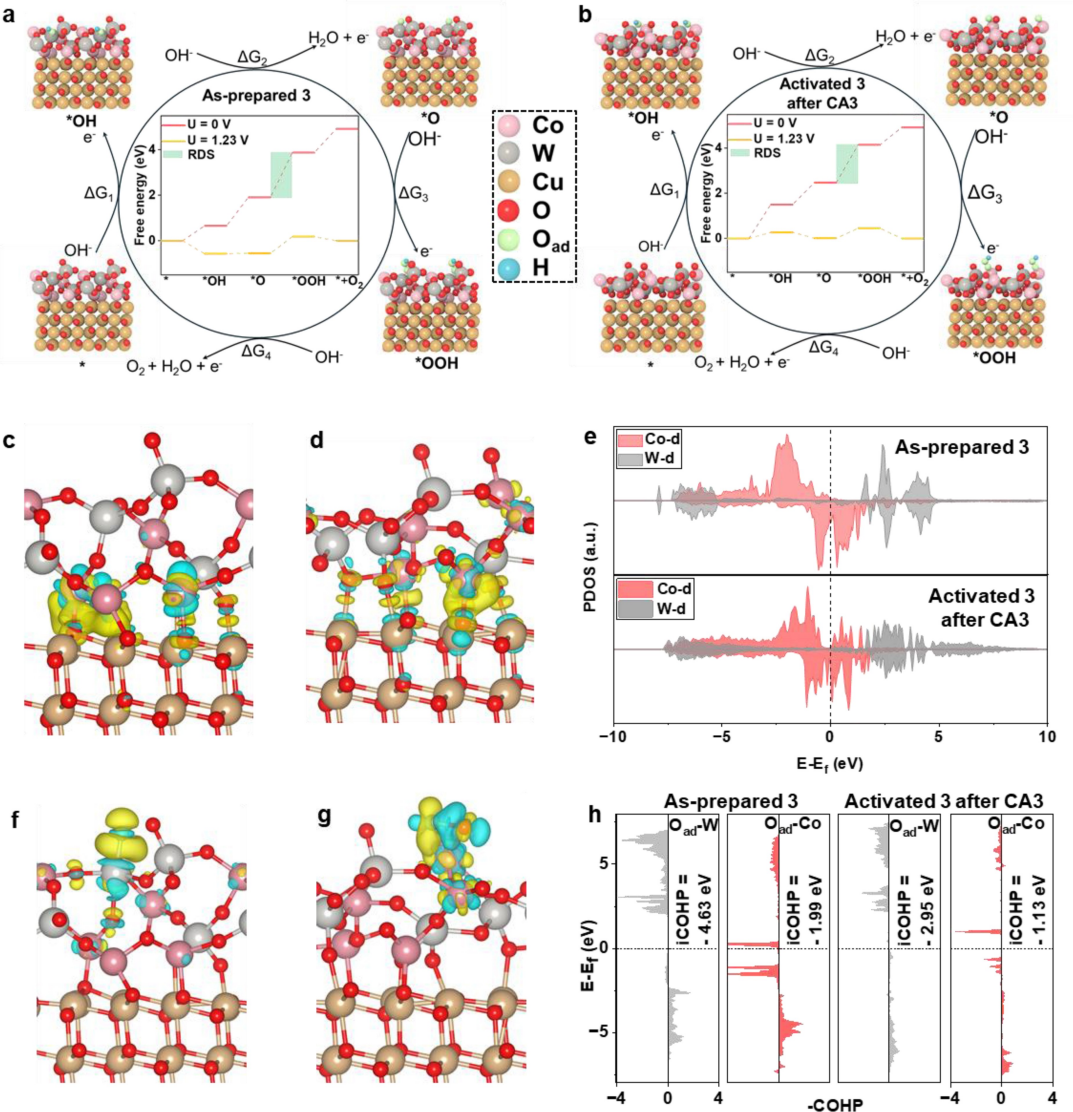
DFT calculations on intrinsic mechanism. Schematic of the whole OER mechanism on as‐prepared **3** (a) and activated **3** after CA3 (b) in the alkaline electrolyte (RDS: rate‐determining step). The inset shows the free energy profile at U=0 V and U=1.23 V. Difference charge density of as‐prepared **3** (c) and activated **3** after CA3 (d). Yellow and cyan regions represent the increase and decrease of electron density, respectively. The iso‐surfaces are plotted at the value of ±0.006 |e|Å^−3^. (e) Projected density of states (PDOS) of d‐orbitals for the as‐prepared **3** and activated **3** after CA3. Difference charge density diagrams of as‐prepared **3** (f) and activated **3** after CA3 (g) after O adsorption on the surface. (h) Crystal orbital Hamilton population (COHP) analyses of O_ad_‐W and O_ad_‐Co bonds of as‐prepared **3** and activated **3** after CA3.

Explicitly, we simulated the adsorption configurations of the reaction intermediates and the OER Gibbs free energy profiles at U=0 V and U=1.23 V, respectively. As evidenced by Figure 5a, all the oxygen‐containing intermediates (*OH, *O and *OOH) adsorbed on the W site of the as‐prepared **3** surfaces, indicative of its preliminary role as the reactive site. Interestingly, for the activated **3** after CA3, the oxygen‐containing intermediates (*OH, *O and *OOH) tended to adsorb on the Co site (Figure 5b), which is considered to be the new active site responsible for the optimized OER performance. Despite the adaptation of adsorption sites, the OER energy profile of both confirmed that the formation of *OOH intermediate from *O is the rate‐determining step (RDS) that overcame the highest energy barrier (Figures 5a, b, **Table S6** and **Table S7**), which was matched well with the experimental ATR‐FTIR and Raman spectroscopy results (Figure 4). Correspondingly, the calculated OER overpotentials (*η*) for the as‐prepared **3** is 0.72 V, while it is 0.44 V for the activated **3** after CA3, which is lower than the calculated overpotential of 0.66 V for the commercial IrO_2_.^[70]^ The significant reduce in calculated overpotential implies increased intrinsic activity after activation. This theoretical finding is in excellent agreement with the self‐optimizing electrochemical performance observed (Figure 3i, **Table S3**), where the experimental overpotential was decreased to 363 mV from 500 mV (at *J*=10 mA/cm^2^).

Additionally, the difference charge density diagrams show that significant charge transfer occurs at the interface between the deposited Co−W oxide and L–CuO substrate, indicative of interfacial interactions witnessed previously by the blue shift of Cu−O vibrational mode (Figure 2e). In comparison to the as‐prepared **3** (Figure 5c), the electron transfer on the interfaces of the activated **3** after CA3 is more homogeneous and pronounced (Figure 5d). Furthermore, the projected density of states (PDOS) was analyzed (Figure 5e) to investigate the electronic properties of the two surfaces. The relative increase in DOS near the Fermi level for the activated **3** after CA3 renders more efficient interactions with the surrounding electrons, complementarily proving the aforementioned enhanced electrical conductivity (displayed in Figure 3h, **Figure S27b** and **Table S3**) and therefore significantly boosted OER performance.^[71]^

To investigate the underlying self‐activation mechanism for OER, we calculated the difference charge density for adsorbed O atom (O_ad_) on the surfaces of as‐prepared **3** (Figure 5f) and activated **3** after CA3 (Figure 5g). Notably, both surfaces exhibit considerable charge transfer as confirmed by Bader charge analysis. Specifically, the O_ad_ bound to W site on the surface of as‐prepared **3** gains 0.88 e , while the O_ad_ bound to the Co site on the surface of activated **3** after CA3 gains 0.31 e .

These results highlight the distinct charge transfer dynamics associated with the self‐optimization process. Next, the crystal orbital Hamilton population (COHP) calculations were conducted under O_ad_‐W or O_ad_‐Co bonding environments, respectively. Note that, the adsorption free energies of O_ad_ on the W site and the Co site on the surface of as‐prepared **3** were determined to be 1.91 eV and 2.52 eV (**Figure S36**), while on the surface of activated **3** after CA3, the adsorption free energies were 2.62 eV and 2.47 eV (**Figure S37**). Here, the integrated COHP value (iCOHP), calculated up to the Fermi level, serves as an effective measure of bonding strength, with more negative iCOHP values indicating a stronger interaction.^[72]^

As depicted in Figure 5h, the iCOHP values for O_ad_‐W and O_ad_‐Co on the surface of as‐prepared **3** were −4.63 eV and −1.99 eV. After CA3, these values decreased to −2.95 eV and −1.13 eV, reflecting a reduction in bond strength. The initially stronger interactions between O_ad_‐W attracted a larger amount of O_ad_ atoms during the early stages of the OER process. However, following the self‐activation process, the incorporation of O_ad_ atoms weakened the adsorption strength. In this context, the moderate bond strength of O_ad_‐Co was obtained, enabling Co as the primary active site. This highlights the crucial role of the W site as the active site precursor, which was adaptive to Co site contributing to the significantly optimized OER catalytic performance. This theoretical finding is fully supported by experimental results especially by W‐oxide free composite **5** with limited OER self‐optimization performance (**Figure S20d** and **Figure S21b**).

## Conclusion

In summary, we have successfully developed highly nanostructured mixed metal oxides based on the lab synthesizable substrate, accessible using a facile wet‐chemical deposition approach. The resulting composite electrocatalyst presents unique and significant self‐optimizing behavior, leading to substantially improved OER performance metrics. Vibrational spectroscopic methods and DFT calculations unravel the dynamic adsorption site of involved intermediates and the nature of true active sites. This work provided essential experimental and theoretical insights into the self‐optimization mechanism of multicomponent composite catalysts and offered a promising approach for the development of robust electrocatalysts for industrial alkaline OER.

## Supporting Information

The authors have cited additional references within the Supporting Information.[^73^, ^74^, ^75^, ^76^, ^77^, ^78^, ^79^, ^80^, ^81^, ^82^, ^83^, ^84^, ^85^, ^86^, ^87^, ^88^, ^89^, ^90^, ^91^]

## Conflict of Interests

The author(s) have declared they have no conflict of interest with regard to this content.

## Supporting information

As a service to our authors and readers, this journal provides supporting information supplied by the authors. Such materials are peer reviewed and may be re‐organized for online delivery, but are not copy‐edited or typeset. Technical support issues arising from supporting information (other than missing files) should be addressed to the authors.

Supporting Information

## Data Availability

The data that support the findings of this study are openly available at Zenodo.org at 10.5281/zenodo.14290873.
