# Supplementary material for: Self‐optimizing Cobalt Tungsten Oxide Electrocatalysts toward Enhanced Oxygen Evolution in Alkaline Media
Source: Angew Chem Int Ed Engl. 2025 Feb 12;64(29):e202424074. doi: 10.1002/anie.202424074 (PMC12258672; doi:10.1002/anie.202424074)
Supplement: Supplementary file 1 — Supporting Information [file ANIE-64-e202424074-s001.pdf]

Angewandte Chemie
Eine Zeitschrift der Gesellschaft Deutscher Chemiker

Supporting Information

Self-optimizing Cobalt Tungsten Oxide Electrocatalysts toward Enhanced Oxygen Evolution in Alkaline Media

C. Nickel, D. L. Troglauer, Z. Dallos, D. Abid, K. Sowa, M. O. Cichocka, U. Kolb, B. Mashtakov, B. F. Mohazzab, S. Han, L. Prädel, L. Ci, D. Li, X. Lin, M. Hua, R. Liu*, D. Gao**

Supporting Information

Self-optimizing Cobalt Tungsten Oxide Electrocatalysts toward Enhanced Oxygen Evolution in Alkaline Media

Christean Nickel,[a] David Leander Troglauer,[a] Zsolt Dallos,[a] Dhouha Abid,[a] Kevin Sowa,[a] Magdalena Ola Cichocka,[b] Ute Kolb,[a, b] Boris Mashtakov,[a] Bahareh Feizi Mohazzab,[a] Shikang Han,[a] Leon Prädel,[c] Lijie Ci,[d] Deping Li,[d] Xiaohang Lin,[e] Minghao Hua,[f,*] Rongji Liu,[a,*] and Dandan Gao[a,*]

[a] M.Sc. C. Nickel, M.Sc. D. L. Troglauer, Dr. Z. Dallos, Dr. Abid, M.Sc. K. Sowa, Dr. U. Kolb, M.Sc. B. Mashtakov, Dr. B. Feizi Mohazzab, B.Sc. S. Han, Dr. R. Liu, Dr. D. Gao
Department of Chemistry, Johannes Gutenberg University Mainz
Duesbergweg 10-14, Mainz 55128, Germany
E-mails: rongji.liu@uni-mainz.de, dandan.gao@uni-mainz.de
[b] Dr. M. O. Cichocka
Research Division of Electron Crystallography, Technical University of Darmstadt
Schnittspahnstr. 9, 64287 Darmstadt, Germany
[c] Leon Prädel
Department for Molecular Spectroscopy, Max Planck Institute for Polymer Research, Ackermannweg 10, 55128Mainz, Germany
[d] Prof. Dr. L. Ci, Prof. Dr. D. Li
State Key Laboratory of Advanced Welding and Joining, School of Materials Science and Engineering
Harbin Institute of Technology (Shenzhen), Shenzhen 518055, People's Republic of China
[e] Prof. Dr. X. Lin
Key Laboratory for Liquid-Solid Structural Evolution and Processing of Materials, Ministry of Education, School of Materials Science and Engineering
Shandong University, Jinan 250061, People's Republic of China
[f] Dr. M. Hua
School of Energy and Power Engineering, Shandong University, Jinan 250061, People's Republic of China
E-mail: huaminghao@mail.sdu.edu.cn

Table of Contents

1. Instrumentation and chemicals

2. Theoretical calculation methods

3. Synthetic section

4. Analytical section

5. Electrochemical studies

6. Theoretical studies

7. References

8. Author Contributions

1. Instrumentation and chemicals

Powder X-ray diffraction (XRD) patterns were recorded on a STADI-P diffractometer (STOE&Cie) equipped with Mo-Kα1 radiation (λ = 0.70930 Å), a Ge (111) monochromator, and a MYTHEN 1K detector (Dectris), in transmission geometry from 10° to 40° in 2θ (step size 0.015°). Room-temperature data were collected on a flat sample holder using acetate-foil for sample preparation.

Scanning electron microscopy (SEM) images and **energy dispersive X-ray spectroscopy (EDX)** analytical data were obtained using a Hitachi 5200 SEM equipped with an EDX detector.

Transmission electron microscopy (TEM) was carried out using a FEI Tecnai F30 S-TWIN at 300 kV equipped with a Gatan US4000 CCD-camera (4096 x 4096 pixels) and a JEOL 2100F operated at 200 kV equipped with a Gatan Ultrascan 1000 2k x 2k CCD-camera. A 150 µm condenser aperture and standard illumination settings were used for TEM images acquisition. Scanning TEM (STEM) and local EDX measurements were performed with a Philips CM 20 TEM (200 kV acceleration voltage) equipped with a EDAX Si-Li EDX detector. Prior to the TEM investigations, the samples were dispersed in ethanol using an ultrasonic bath and sprayed on a carbon-coated Au /Cu grid using an ultrasonicator.

Attenuated total reflection Fourier transform infrared spectroscopy (ATR-FTIR) was carried out on a Bruker Alpha II equipped with a PIKE Miracle Diamond ATR unit.

Raman spectroscopy was performed on a Renishaw Raman spectrometer using a laser excitation wavelength of 532 nm.

Thermogravimetric analysis (TGA) was performed by METTLER TOLEDO®. Analysis of the sample was carried out on a TGA 2 STARe system under N_2 flow with a flow rate of 60 mL/min. A crucible made of polycrystalline aluminium oxide (PCA/Saphir) was used in a temperature range of 25 - 1000 °C. A heating rate of 10 °C/min was applied.

X-ray photoelectron spectroscopy (XPS) measurements were performed with monochromatized Al Kα exciting X-radiation using a PHI Quantera SXM system. The binding energies were calibrated based on C 1s (284.8 eV)

Inductively coupled plasma optical emission spectrometry (ICP-OES) was performed on a Perkin Elmer Plasma 400 spectrometer. All measurements were conducted in aqueous HNO_3 solution.

Nitrogen adsorption-desorption isotherms were measured at 77 K using a 3P Micro 300 Surface area and Pore Size Analyzer instrument. The experiment was carried out under vacuum conditions 20 h at 60°C (333 K), with nitrogen gas serving as the adsorbate. The pore size distribution was determined using the Barrett-Joyner-Halenda (BJH) method, incorporating a KJS correction.

Contact angle measurements were performed using the sessile drop method using 1.0 M KOH electrolyte solution.

Chemicals: Ammonium peroxosulfate (APS), (Honeywell, CAS No. 7727-54-0), sodium hydroxide (ROTH, CAS No. 1370-73-2), Copper oxide (ACROS Organics, CAS No. 1317-38-0), Cobalt(II) oxide (Alfa Aesar, CAS No. 1307-96-6), Tungsten (VI) Oxide (Acros Organics, CAS No. 1314-35-8), Sodium tungstate dihydrate (VWR, CAS No. 10213-10-2), Sodium metasilicate pentahydrate (VWR, CAS No. 10213-79-3), Potassium chloride (99.0%-100.5%, Carl Roth, CAS No. 7447-40-7), Cobalt (II) nitrate hexahydrate (99%, VWR, CAS No. 10026-22-9), Potassium hydroxide (Carl Roth, CAS No. 1310-58-3), Potassium nitride (VWR, CAS No. 7757-79-1), Hydrochloric acid (37%, VWR, CAS No.7647-01-0). Commercial carbon paper (CAS No. 7782-42-5) with a specific dimension of 0.2 x 190 x 190 mm^3 was purchased from Alfa Aesar GmbH & Co. KG, which was then cut into 0.2 x 10 x 30 mm^3. Before using as substrate, the carbon paper was cleaned with acetone, deionized water, and ethanol, respectively (immersion time: 15 min per solvent). All chemicals were used as received.

2. Theoretical calculation methods

2.1 Density functional theory calculations

All the density functional theory (DFT) calculations were performed with the Projector Augmented Wave (PAW) pseudopotentials in the Vienna ab initio Simulation Package (VASP).[1,2] The electron exchange-correlation energy was approximated by the Perdew-Burke-Ernzerhof (PBE) functional within the generalized gradient approximation (GGA).[3] The kinetic cutoff energy was set to 520 eV. The convergence criteria for the self-consistent field (SCF) step was 1 × 10^{-5} eV. Structure optimizations were executed until the force was less than 0.02 eV/Å. Spin polarization was considered in all calculations. The solvation effect was drawn into the adsorbed free energy of diverse intermediates via a polarized continuum model in VASPsol.[4] The dielectric constant of the solvent was set to 80, which corresponds to that of water at room temperature.

To approach the realistic amorphous Co-W oxide structure for the insights into the OER mechanism, the amorphous "as-prepared **3**" and "activated **3** after CA3" slab models were obtained using AIMD simulations and the melt-quenched method as prior research.[5,6]

Firstly, building on the experimental XRD, TEM, XPS and ICP-OES data, the as-prepared **3** slab was built with formula of $Co_5W_4Cu_{32}O_{48}$, and the activated **3** after CA3 slab was built with formula of $Co_5W_4Cu_{32}O_{52}$, where the subscripts denote the actual atom counts in the slab models. The optimized CuO (111) slabs were interfaced with the Co-W oxide slabs (as-prepared **3** and activated **3** after CA3) with a distance of 2 Å and fully relaxed by DFT at 0 K. Then the heterostructures were melted and equilibrated at 1200 K for 14 ps to remove the memory effect from the initial configuration.

Secondly, the heterostructures were rapidly cooled down to 300 K for 10 ps with a cooling rate of 90 K/ps. The total duration of the AIMD simulation reached 24 ps. The time interval was set to 2 fs during all the AIMD simulation. The Nosé-Hoover thermostat was employed with "SMASS = 2" in VASP.

Finally, the last trajectories were further optimized by DFT calculation to release the atomic forces and get the amorphous slabs in 0 K configurations. These amorphous slabs were then used for subsequent free energy change analyses. The slab models consist of six atomic layers, with the bottom three layers fixed during geometry optimization while the other layers and adsorbates were allowed to relax. All the slab models were separated by a vacuum depth of 15 Å. The Brillouin was set with a Γ-centered k-point grid of 3 × 3 × 1.

2.2 Oxygen evolution reaction mechanism

The OER reaction pathways are required as following:

Step 1 (ΔG_1): $* + OH^- \rightarrow * OH + e^-$
Step 2 (ΔG_2): $* OH + OH^- \rightarrow * O + H_2O + e^-$
Step 3 (ΔG_3): $* O + OH^- \rightarrow * OOH + e^-$
Step 4 (ΔG_4): $* OOH + OH^- \rightarrow * + O_2 + H_2O + e^-$
where $*$ denotes the adsorption site on the slabs for oxygen-containing intermediates.

The computational hydrogen electrode (CHE) model was adopted to calculate the adsorption-free energies of the OER mechanism.[7] The Gibbs free energy for each elementary step was calculated via following equation:

$$\Delta G = \Delta E + \Delta ZPE + \Delta \int C_P \, dT - T\Delta S$$

where ΔE is the total energy difference of the reaction calculated by DFT, ΔZPE is the zero-point energy (ZPE) change, $\Delta \int C_P \, \mathrm{d}T$ is the enthalpy thermal correction the difference in integrated heat capacity from 0 to 298.15 K, $T\Delta S$ is the entropy change at a reaction temperature at T = 298.15 K.[8] The ΔZPE, $\Delta \int C_P \, \mathrm{d}T$ and $T\Delta S$ corrections were calculated from vibrational frequencies that were

obtained by diagonalizing the Hessian matrix with respect to the three Cartesian degrees of freedom of each atom of the adsorbed intermediates. Low frequency modes were reset to a threshold value of 50 cm^{-1} to avoid a spurious contribution.[9] It is important to note that the theoretical overpotential is independent of pH or potential values because the free energies vary uniformly with pH and U, ensuring that the potential-determining step remains consistent. Therefore, the free energy profile was conducted under standard conditions (pH = 0, T = 298.15 K) with U = 0. The theoretical thermodynamic limiting potential (U_L) and overpotential (η) of OER, which can be the measurement of the activity of a catalyst, are defined as follows:

$$U_L = -\{\Delta G_1 / e, \Delta G_2 / e, \Delta G_3 / e, \Delta G_4 / e\}_{\max}$$

$$\eta = U_L - 1.23V$$

Moreover, the theoretical overpotential η serves as an indicator of catalyst activity and cannot be directly compared to experimentally measured overpotentials, which are inherently influenced by current density.[10,11]

All thermodynamic data were processed with VASPKIT code.[12] The Bader charge analysis method was used to calculate the atomic charge.[13] The Lobster software was used to perform the crystal orbital Hamilton population (COHP) analysis to obtain the bonding and anti-bonding information.[14]

3. Synthetic section

3.1 $K_8[\alpha\text{-}SiW_{11}O_{39}]\cdot 13H_2O$

The compound was synthesized according to the published procedure.[15,16] Purity was confirmed by ATR-FTIR (characteristic bands are given as wavenumbers in cm^{-1} using the following abbreviations: s, strong; m, medium; w, weak): 3,389 (s), 1624 (s), 992 (m), 954 (s), 869 (s), 788 (m), 707 (s), 497 (m), and 463 cm^{-1} (w).

3.2 Lab-synthesized CuO (L-CuO) substrate

The L-CuO was clollected as the precipitate from our previous work for bulk nanostructuing of Cu foam.[17] In brief, cleaned Cu foam (Dimension: 2 x 10 x 40 mm^3, 90 % porosity, Xiamen Tmax Battery Equipments Limited, China) was immersed into 15 mL oxidizing aqueous solution (0.133 M APS, 2.66 M NaOH) at room temperature for 15 h, from which a Janus-type nanostructure Cu foam was formed. Meanwhile, the black CuO precipitate in the reaction vessel was obtained and collected subsequently.

3.3 Co-W oxides deposited on L-CuO and commercial CuO (C-CuO)

The composites are prepared by a facile one-step hydrothermal deposition process. Initially, the as-collected L-CuO was dispersed in a 100 mL Teflon autoclave liner containing a precursor solution of $Co(NO_3)_2\cdot 6H_2O$ and $K_8[SiW_{11}O_{39}]\cdot 13H_2O$ in 40 mL water, where different Co^{2+} / $[SiW_{11}O_{39}]^{8-}$ molar ratios (1 : 1, 2 : 1, 3 : 1, 4 : 1) were used. The mixture was then heated in an autoclave at 180 °C for 8 h under autogenous pressure. After cooling to room temperature, the obtained mixed metal oxides were collected, rinsed with deionized H_2O and ethanol, and subsequently air-dried. The final products obtained from Co^{2+} / $[SiW_{11}O_{39}]^{8-}$ molar ratios of 1 : 1 (precursor 1), 2 : 1 (precursor 2), 3 : 1 (precursor 3) and 4 : 1 (precursor 4) were designated as composites **1, 2**, **3** and **4**, respectively. To elucidate the role of the individual components in precursor 3, the synthesis was carried out in an identical fashion but in the presence of only Co^{2+} (precursor 5 resulting in composite **5**) or $[SiW_{11}O_{39}]^{8-}$ (precursor 6 resulting in composite **6**). To explore the role of CuO substrate, C-CuO was employed as the reference support for the identical deposition process in precursor 3 (Co^{2+} / $[SiW_{11}O_{39}]^{8-}$ molar ratio of 3 : 1), leading to composite **7**.

Table S1. Components of precursor solutions for hydrothermal deposition process.

	Precursor 1	Precursor 2	Precursor 3	Precursor 4	Precursor 5	Precursor 6	Precursor 3
	Composite **1**	Composite **2**	Composite **3**	Composite **4**	Composite **5**	Composite **6**	*Composite **7**
Co^{2+} / $[SiW_{11}O_{39}]^{8-}$	1 : 1	2 : 1	3 : 1	4 : 1	-	-	3 : 1
Co^{2+}	1 mmol	2 mmol	3 mmol	4 mmol	3 mmol	-	-
$[SiW_{11}O_{39}]^{8-}$	1 mmol	1 mmol	1 mmol	1 mmol	-	1 mmol	-

* : C-CuO as the substrate

3.4 Working electrode preparation

The respective composite (**1**, **2**, **3**, **4**, **5**, **6**, **7**) (10 mg) was dispersed in a mixture of 780 μL of ethanol, 200 μL of deionized H_2O, and 20 μL of 5.0 wt % Nafion, followed by sonication for ca. 120 min to form a homogeneous suspension. The above prepared suspension (60 μL) was drop-casted onto cleaned carbon paper (controlled catalyst-loaded area: 1 cm^2), giving a catalyst loading of 0.6 mg/cm^2 (based on the geometric surface area). After being dried, the catalyst-coated carbon paper was further modified with a thin film of Nafion by drop-casting 20 μL of 0.5 wt % Nafion solution (in isopropanol).

3.5. Electrochemcial measurements

Electrochemical measurements were performed on a CH Instruments CHI 760E workstation in a two-compartment electrochemical cell seperated by a proton exchange membrane (Nafion 117) with three-electrode configuration (working electrode: CP coated with the electrocatalyst, reference electrode: mercury / mercury oxide electrode, counter electrode: graphite rod) in Ar purged 1.0 M KOH electrolyte (pH 13.8, 70 mL for both compartments). Before use, the Nafion 117 membrane was pretreated by slightly boiling (at 80 °C) in 5 wt.% H_2O_2 solution, distilled water, 0.1 M H_2SO_4 solution and distilled water, respectivey (treatment time: 1 h per solution).

All electrodes were per-conditioned by 40 cyclic voltammetry (CV) sweeps (at 100 mV/s) between 0.2 V to 0.5 V vs RHE at room temperature. Polarization curves for OER were recorded by linear sweep voltammetry (LSV) with a scan rate of 5 mV/s with 90% *iR* compensation. All potentials were converted to the reversible hydrogen electrode (RHE) according to the Nernst equation ($E_{RHE} = E_{Hg/HgO} + E^0_{Hg/HgO} + 0.059$ V x pH, where $E^0_{Hg/HgO}$ is the standard potential of 0.098 V for mercury / mercury oxide electrode).

The electrochemically active surface area (ECSA) was examined by the electrochemical double-layer capacitance analysis of the catalyst, which was determined from the CV curves under an incremental scanning rate from 20 to 120 mV/s, in the potential window from 1.08 to 1.18 V vs RHE. The electrochemical impedance spectroscopy (EIS) was performed using AC impedance spectroscopy measured at 1.63 V vs RHE, and the frequency ranged from 100 kHz to 0.1 Hz.

4. Analytical section

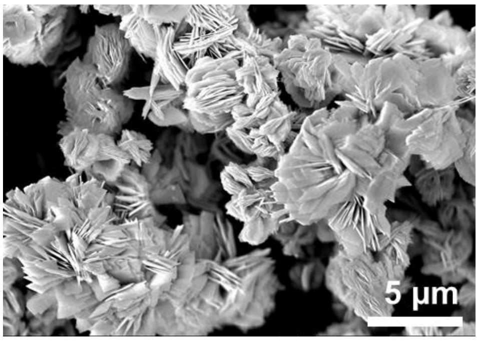

Fig. S1. SEM image of L-CuO in low magnification.

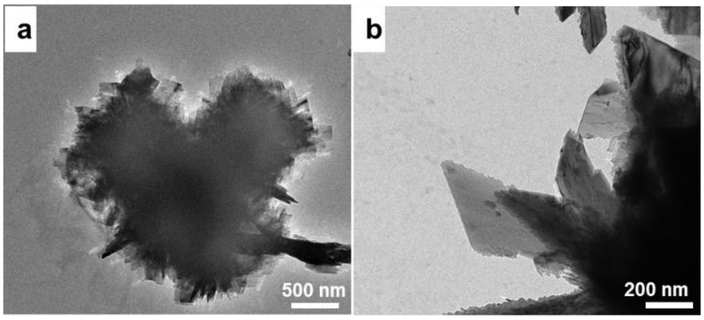

Fig. S2. TEM images of L-CuO in different magnifications.

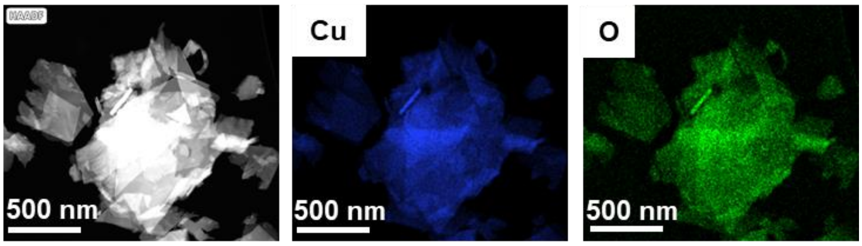

Fig. S3. HAADF-STEM image and corresponding elemental mappings of L-CuO.

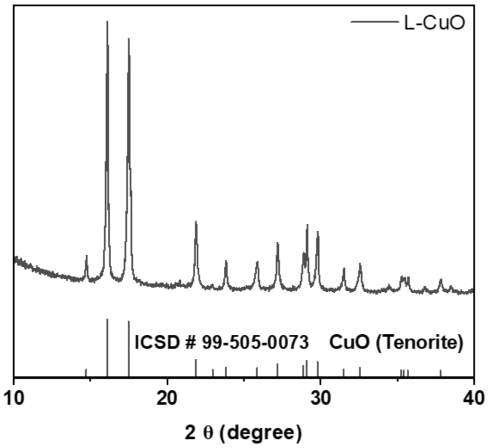

Fig. S4 pXRD of L-CuO substrate.

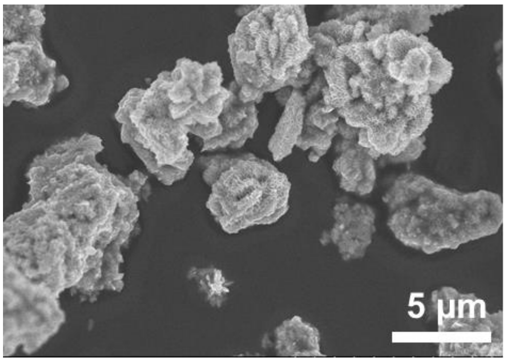

Fig. S5. SEM image of **3** in low magnification.

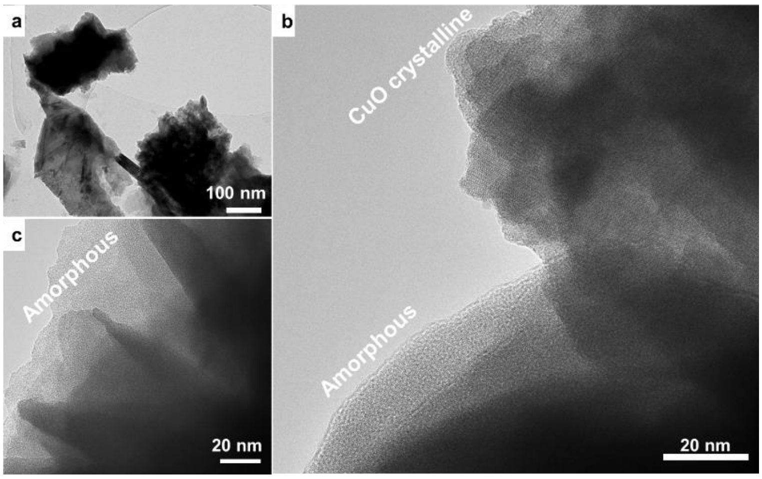

Fig. S6. TEM images of **3** in different magnifications.

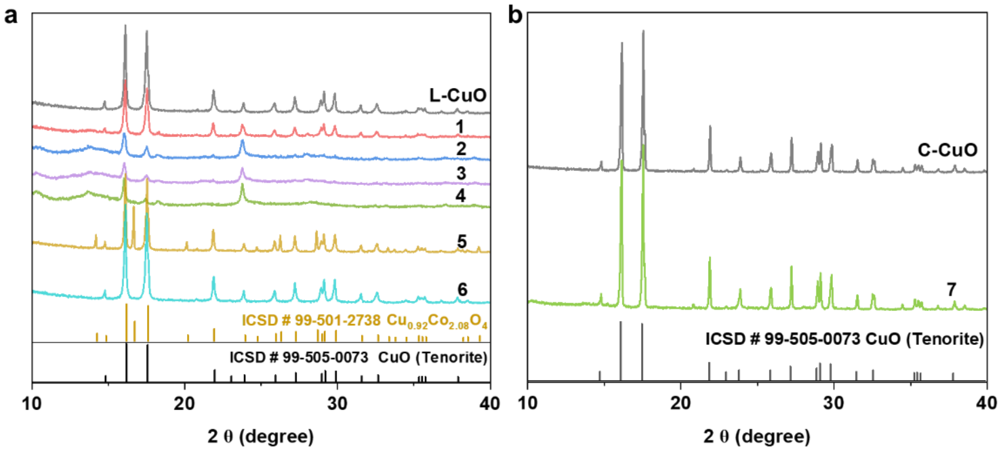

Fig. S7 pXRD of (a) **1** - **6** and L-CuO, (b) **7** and C-CuO.

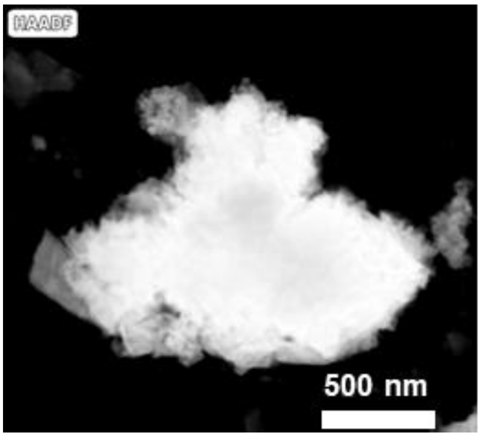

Fig. S8. (a) HAADF-STEM image of **3** for the elemental mapping shown in **Fig. 1i**.

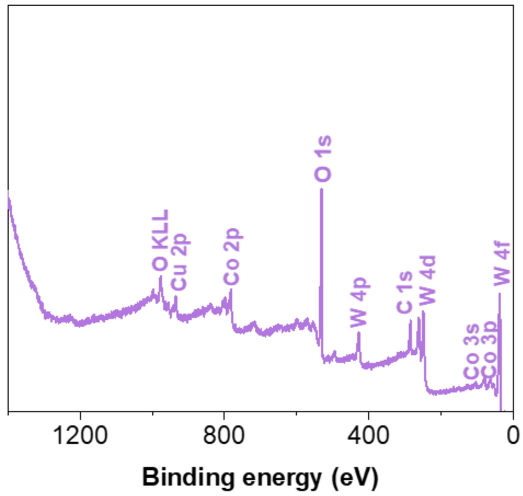

Fig. S9. XPS survey spectrum of **3**.

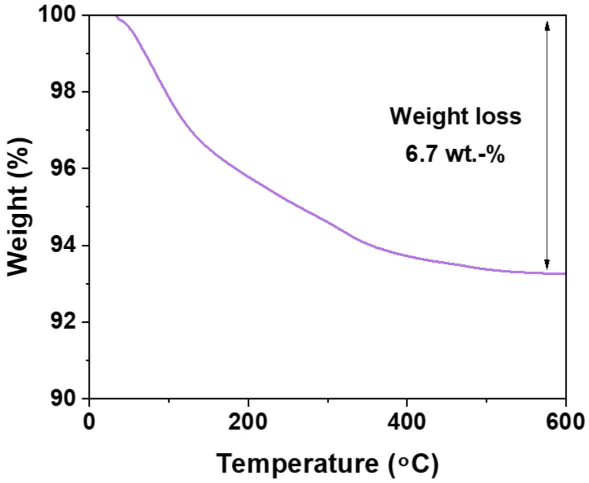

Fig. S10. Thermogravimetric analysis of **3**. Weight loss of 6.7 wt.% was observed between 30 to 600 °C due to the loss of lattice water ligands.

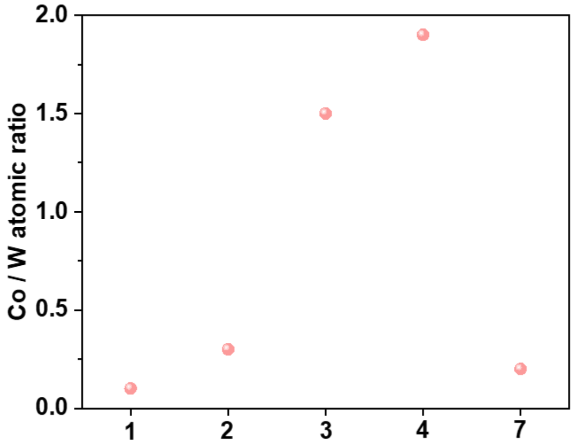

Fig. S11. Co / W atomic ratio of **1**, **2**, **3**, **4** and **7** based on ICP-OES analyses.

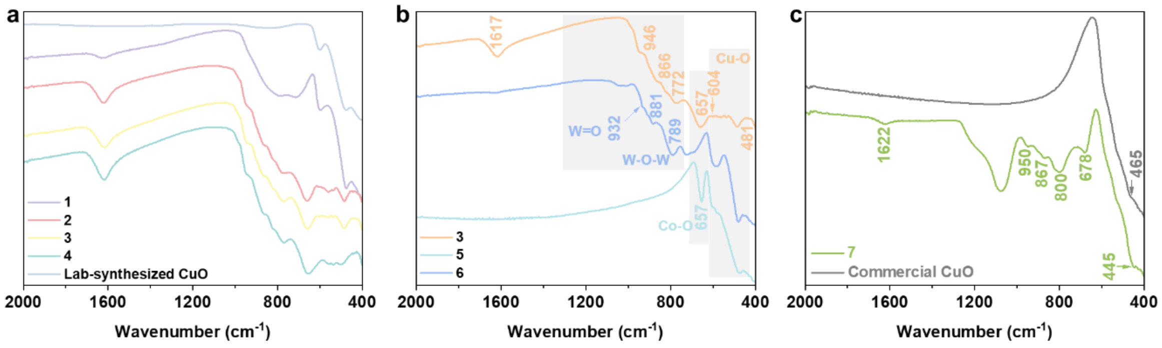

Fig. S12. ATR-FTIR spectra of **1** - **7**, L-CuO and C-CuO.

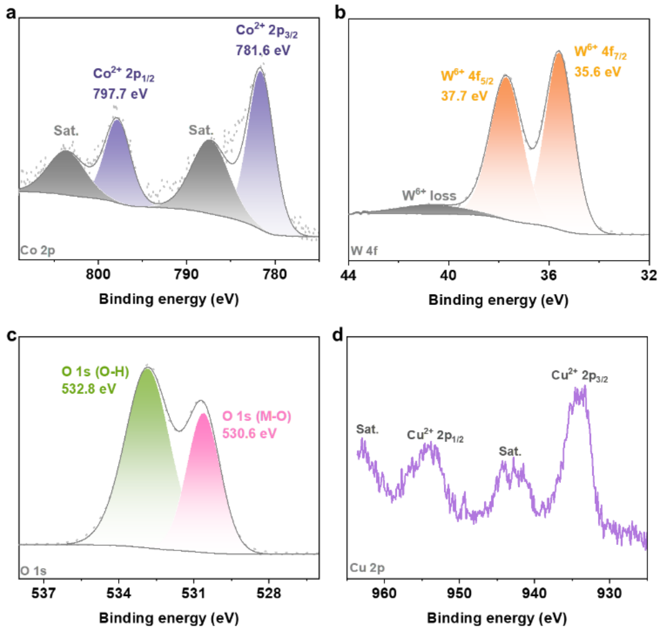

Fig. S13. XPS spectra for (a) Co 2p, (b) W 4f, (c) O 1s and (d) Cu 2p of **7**.

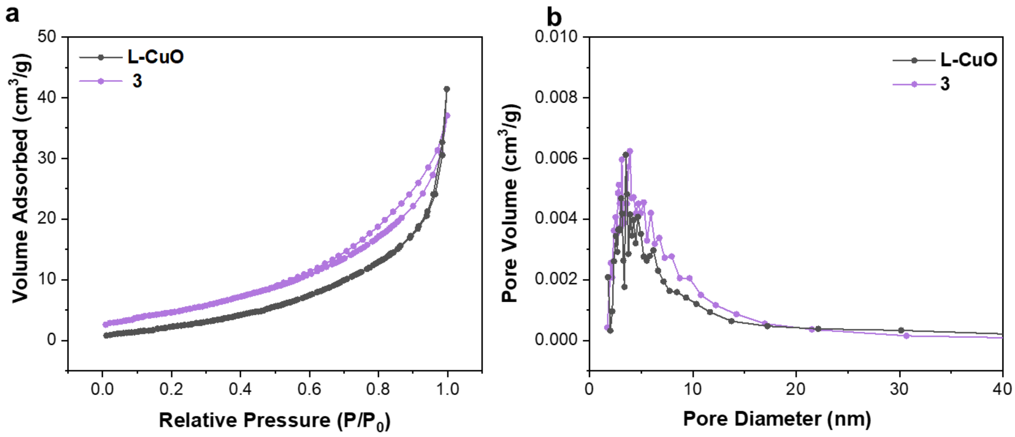

Fig. S14. (a) N_2 adsorption–desorption isotherms and (b) pore size distribution curves of L-CuO and **3**.

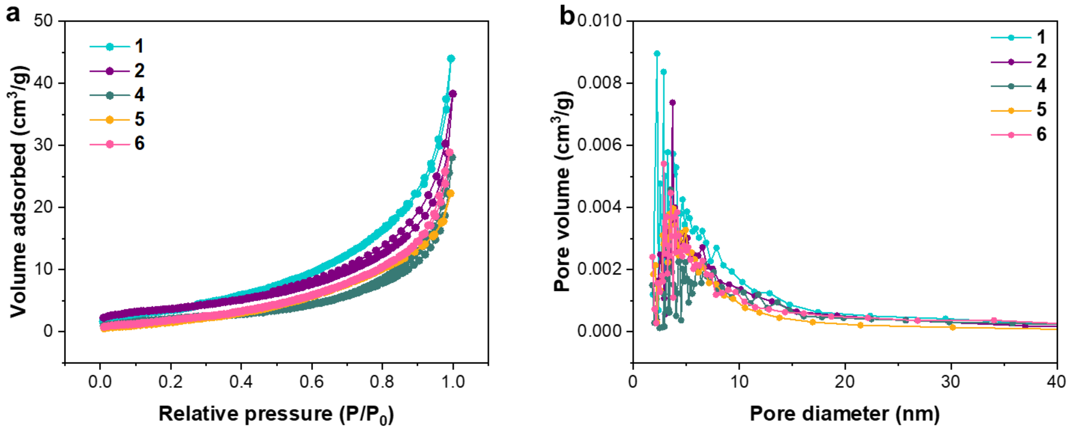

Fig. S15. (a) N_2 adsorption-desorption isotherms and (b) pore size distribution curves of **1**, **2**, **4**, **5** and **6**.

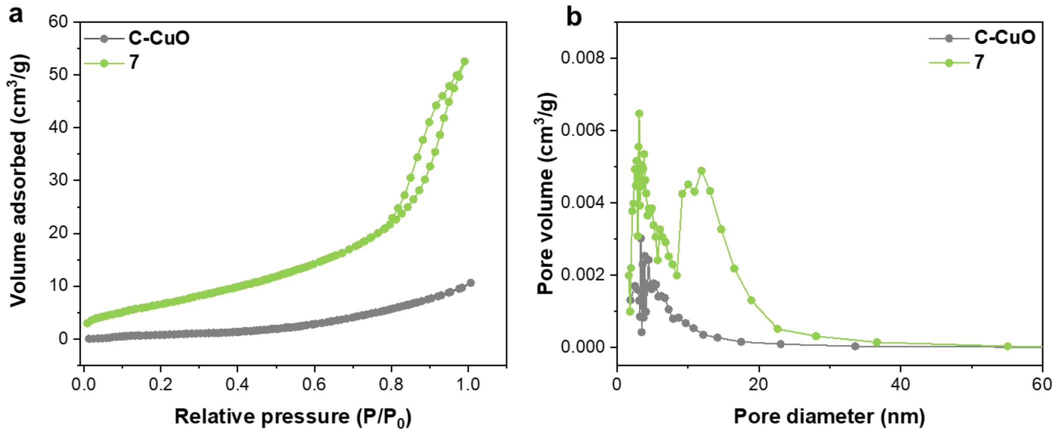

Fig. S16. (a) N_2 adsorption–desorption isotherms and (b) pore size distribution curves of C-CuO and **7**.

Table S2. Specific surface area and pore size distribution for the L-CuO, C-CuO and as-prepared **1** - **7**.

Material	Surface area (S_{BET}) m^2/g	Pore volume (V_{Tot}) cm^3/g	Pore size (BJH) nm
L-CuO	11.156	0.052	11.424
As-prepared **1**	14.785	0.063	17.135
As-prepared **2**	13.482	0.049	14.672
As-prepared **3**	18.379	0.054	16.322
As-prepared **4**	8.337	0.037	13.588
As-prepared **5**	5.359	0.033	15.287
As-prepared **6**	8.210	0.045	16.462
C-CuO	5.289	0.015	11.638
As-prepared **7**	26.505	0.081	12.232

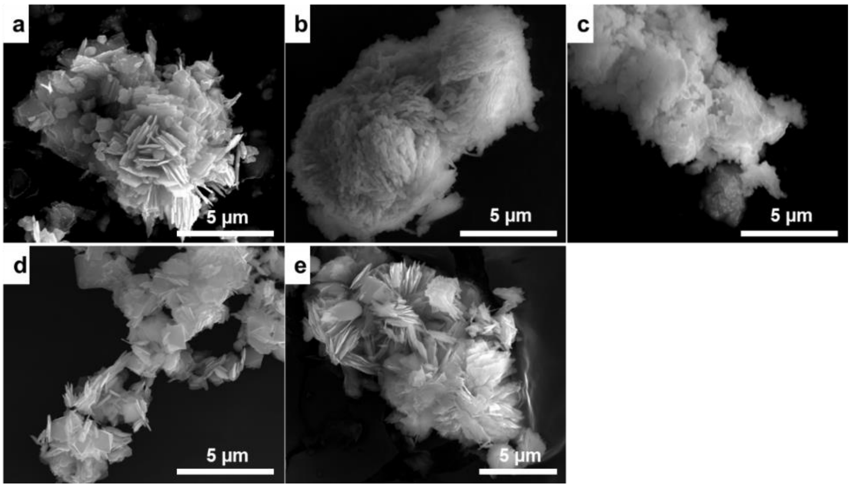

Fig. S17 SEM images of **1** (a), **2** (b), **4** (c), **5** (d) and **6** (e).

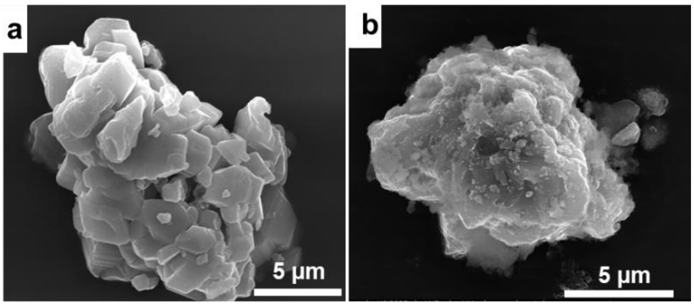

Fig. S18 SEM images of C-CuO (a) and **7** (b).

5. Electrochemical studies

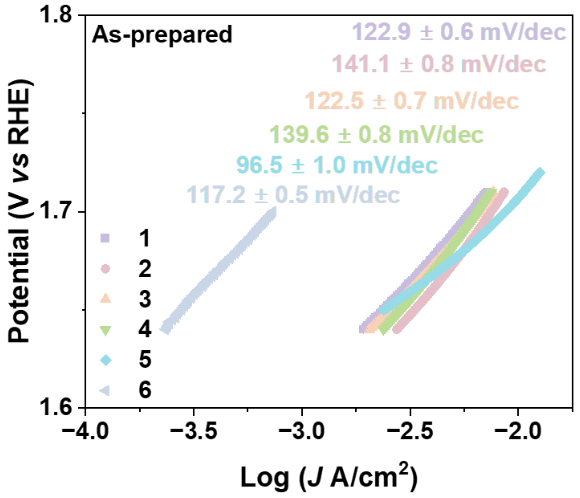

Fig. S19 Tafel slopes of as-prepared **1** - **6** (derived from LSV curves in **Fig. 3a**).

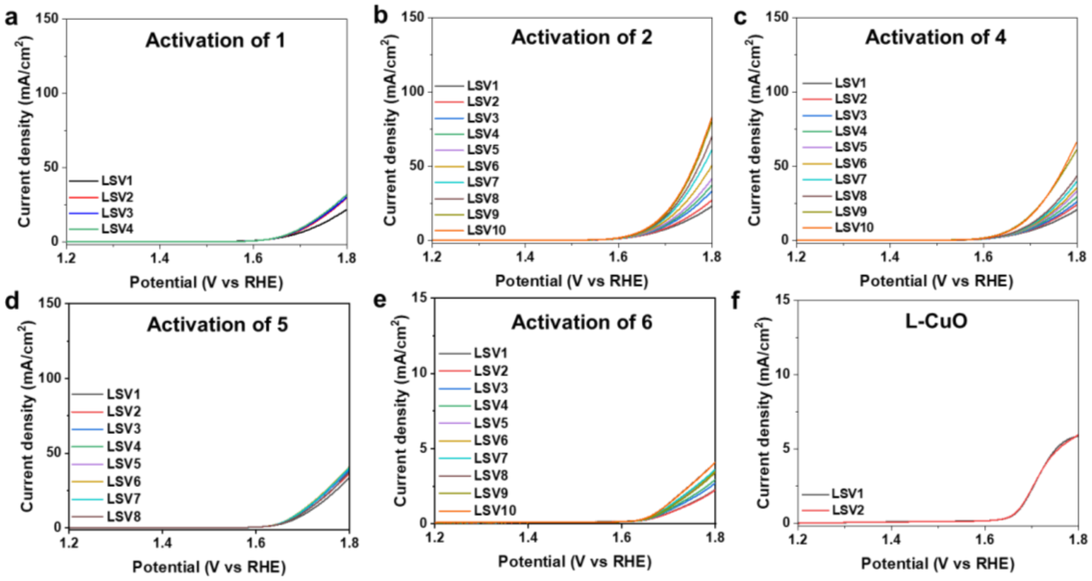

Fig. S20. Successive LSV scanning for **1** (a), **2** (b), **4** (c), **5** (d), **6** (e) and L-CuO (f).

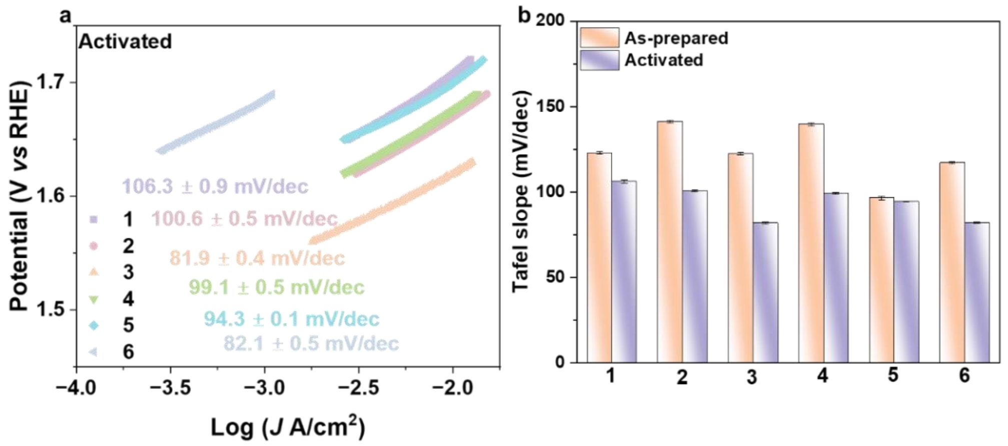

Fig. S21. (a) Tafel slopes of activated **1** - **6** (derived from LSV curves in **Fig. 3c**). (b) Tafel slopes comparison between as-prepared and activated **1** - **6**.

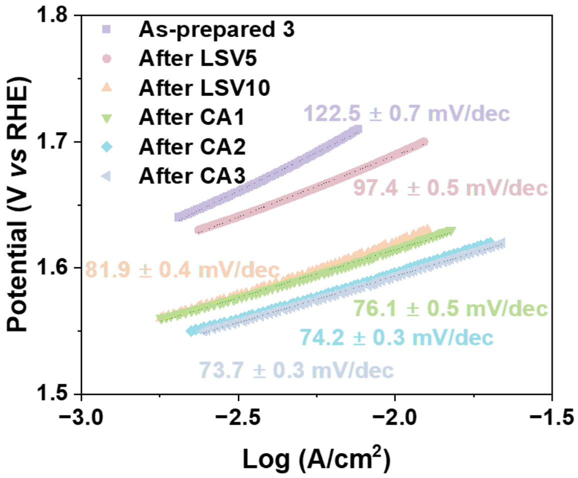

Fig. S22. The evolution of Tafel slopes of **3**.

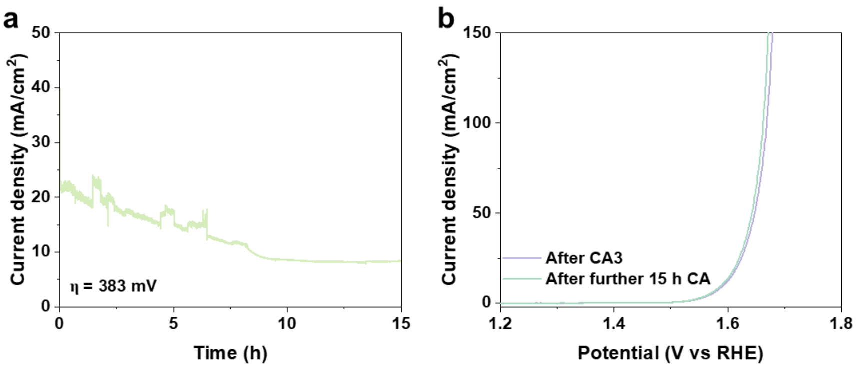

Fig. S23. (a) 15 h CA measurement of **3** after CA 3, (b) LSV comparison before and after 15 h CA.

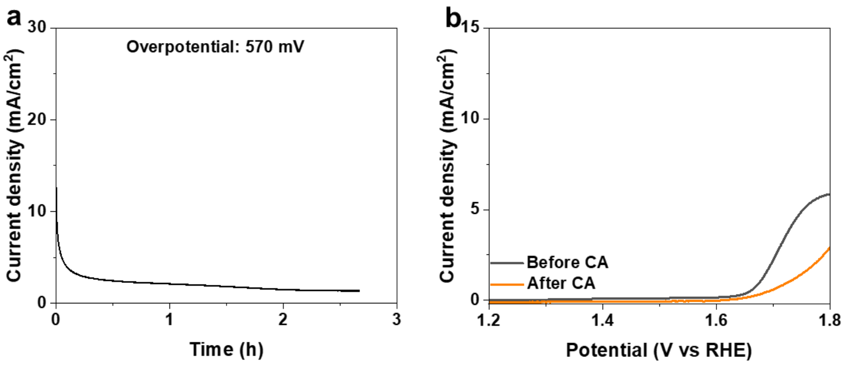

Fig. S24. (a) CA measurement (< 3 h) and (b) LSV comparison before and after CA for L-CuO.

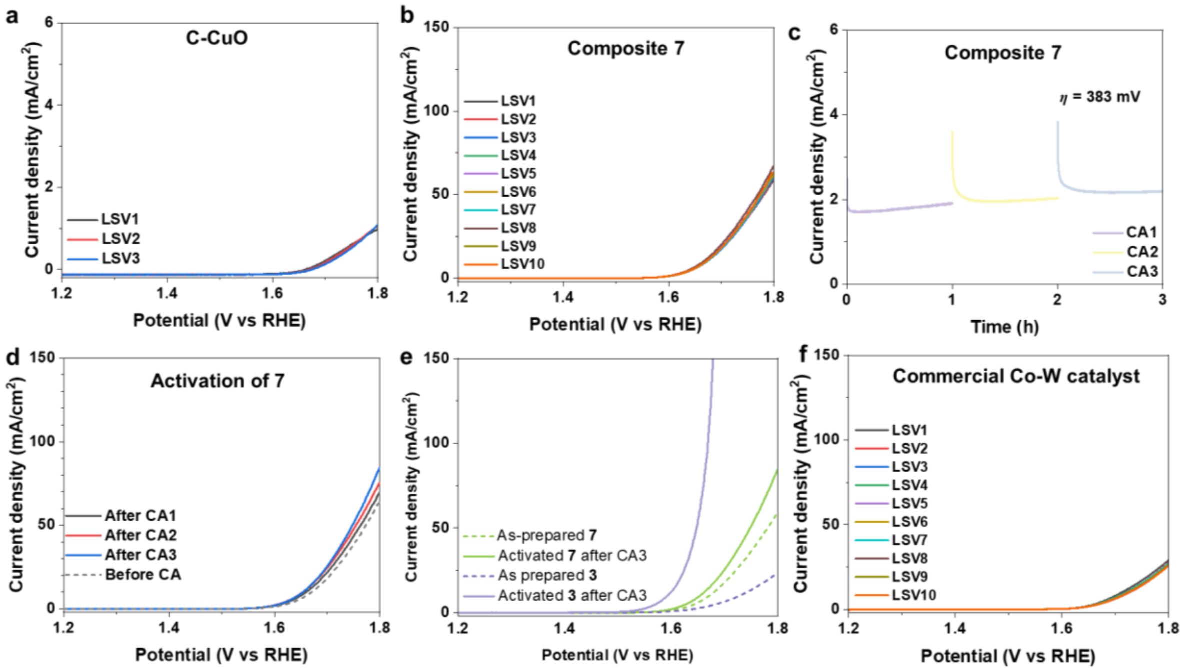

Fig. S25. Successive LSV scanning for C-CuO (a), and **7** (b). (c) Stepped chronoamperometry (CA) test of **7** over a period of 3 h. (d) LSV curves of activated **7** before and after CA tests. (e) LSV comparison of activation of **3** and **7**. (f) Successive LSV scanning for commercial Co oxide and W oxide catalyst mixed with L-CuO (Co / W atomic ratio = 1.5 : 1).

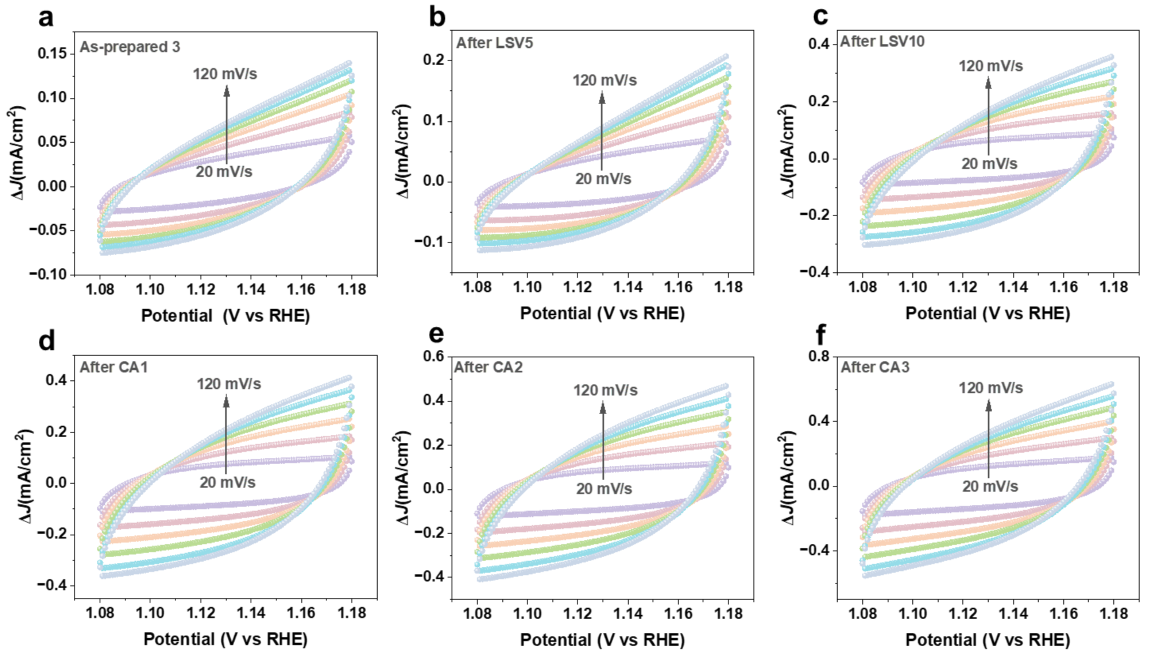

Fig. S26. Electrochemical capacitance measurements to determine the evolution of ECSA of **3**: (a) As-prepared, (b) after LSV5, (c) after LSV10, (d) after CA1, (e) after CA2 and (f) after CA3.

ECSA is calculated based on the following equations:

(1) $\Delta J = v \times 2 \times C_{\mathrm{dl}}$

(2) ECSA = $C_{\mathrm{dl}} / C_{\mathrm{s}}$

(3) Cs = 40 μF/cm^2 per cm^2

where v is scanning rate, ΔJ (= J_a - J_c) is anodic and cathodic current density differences extracted at 1.13 V vs RHE. C_{dl} is double-layer capacitance and C_s is the specific capacitance of the catalyst.[18] The ECSAs of **3** during studied OER process are depicted in **Fig. S27a**, and given in **Table S3**.

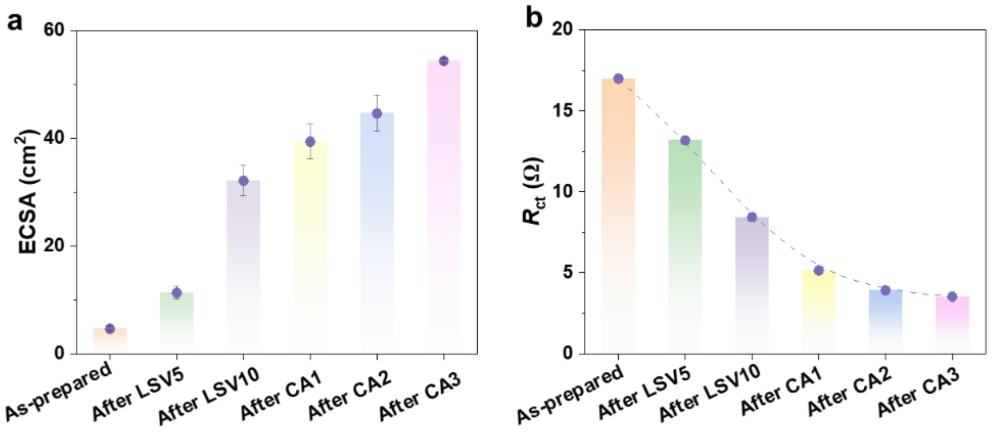

Fig. S27. The evolution of (a) ECSA and (b) R_{ct} of composite **3**.

Table S3. Self-optimization of **3** during studied OER process.

	η (mV)		J	Tafel slope	ECSA	R_{ct}
	J = 10 mA/cm^2	J = 25 mA/cm^2	(mA/cm^2) η = 450 mV	(mV/dec)	(cm^2)	(Ω)
As-prepared	500	> 570	4.6	122.5 ± 0.7	4.6 ± 0.4	17.0
LSV5	460	510	8.2	97.4 ± 0.5	11.3 ± 1.3	13.2
LSV10	390	426	47.2	81.9 ± 0.4	32.1 ± 2.9	8.4
CA1	385	420	57.3	76.1 ± 0.5	39.4 ± 3.3	5.1
CA2	367	398	149.4	74.2 ± 0.3	44.6 ± 3.4	3.9
CA3	363	394	149.6	73.7 ± 0.3	54.4 ± 0.4	3.6

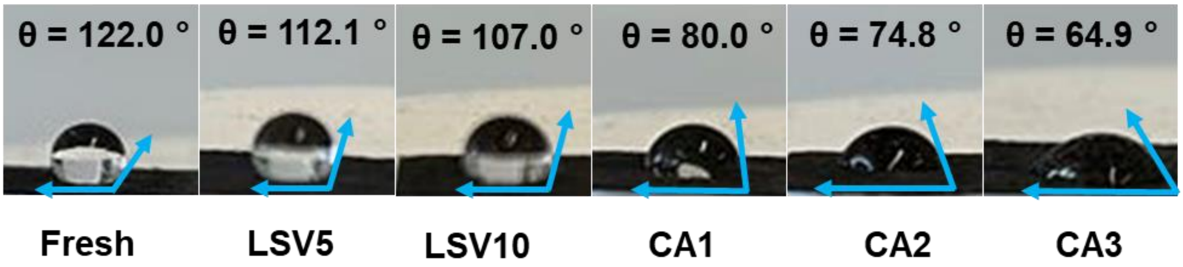

Fig. S28. Sessile drop analysis of the evolution of contact angle between a 1.0 M aqueous KOH droplet and the electrode surface (**3** coated carbon paper).

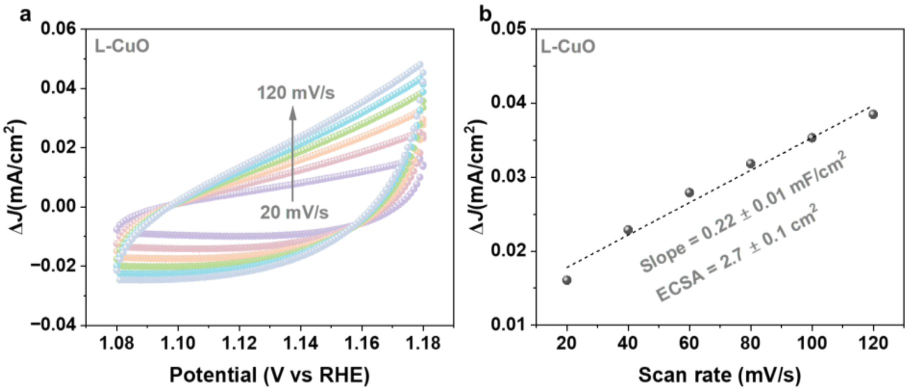

Fig. S29. (a) Electrochemical capacitance measurements to determine the ECSA of L-CuO and (b) Measured capacitive currents plotted as a function of scan rate.

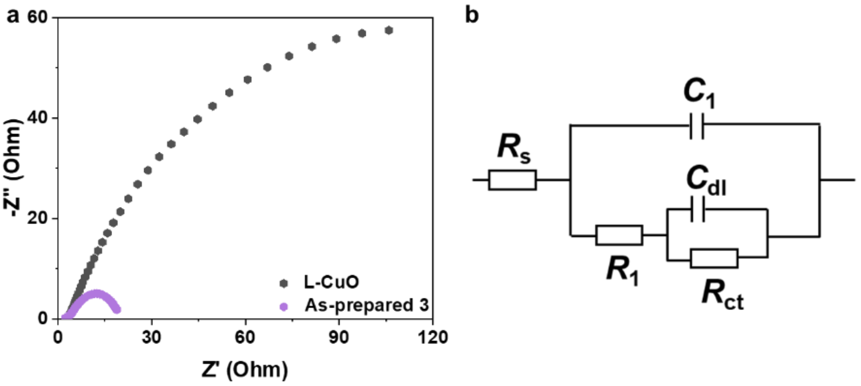

Fig. S30. (a) EIS (Nyquist plots) comparison of (a) L-CuO and as-prepared **3**. (b) Equivalent circuit model fitted using ZView software. (R_s: solution resistance, R_1 / C_1: additional contact impedance / capacitance, R_{ct}: charge transfer resistance, C_{dl}: double layer capacitance).

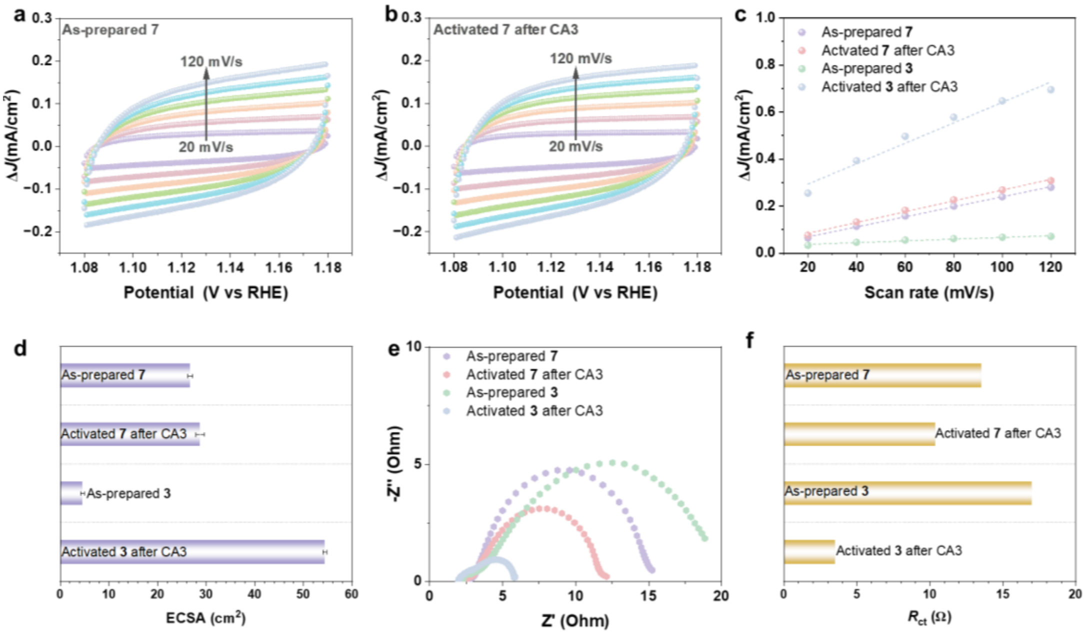

Fig. S31. Electrochemical capacitance measurements to determine the ECSA of (a) as-prepared **7** and (b) activated **7** after CA3. Comparison of (c) measured capacitive currents plotted as a function of scan rate, (d) ECSA, (e) EIS (Nyquist plots) and (f) R_{ct} of **3** activation and **7** activation.

Table S4 Comparison of catalytic performance of activated **3** and reported Co-W oxide based OER electrocatalysts

Catalyst @ support	Overpotential (mV)[a]	Tafel slope (mV/dec)	ECSA (cm^2)	R_{ct} (Ω)	Self-optimization	Time required for complete optimization (h)	Electrolyte KOH (M)	Ref.
$Co_{1.5}WO_{4.3}$@CuO · xH_2O	363	73.7 ± 0.3	54.4 ± 0.4	3.6	Yes	3	1	This work
Mixed Co-W oxide@TiO_2	490	60 ± 0.5	4.25 ± 0.075	10.44	Yes	10	0.1	[19]
W:CoO (s)	320	45	2.09	N.A.	Yes	2	0.1[b]	[20]
$CoWO_4$	450	82	N.A	N.A.	No	N.A.	1	[21]
$Co_{0.5}Fe_{0.5}WO_4$	331	36.8	28.6	N.A.	No.	N.A.	1	[22]
Mixed Co-Cu-W oxide@Cu foam	313	162	275 ± 7	13.0	No	N.A.	0.1	[23]
W-$CoMoO_4$	680	106	N.A.	N.A.	No	N.A.	0.1	[24]
$[Co_{6.8}Ni_{1.2}W_{12}O_{42}(OH)_4(H_2O)_8]$@Ni foam	360	126	N.A.	N.A.	No	N.A.	0.1	[25]

[a] J = 10 mA/cm^2

[b] Electrolyte: 0.1 M NaOH

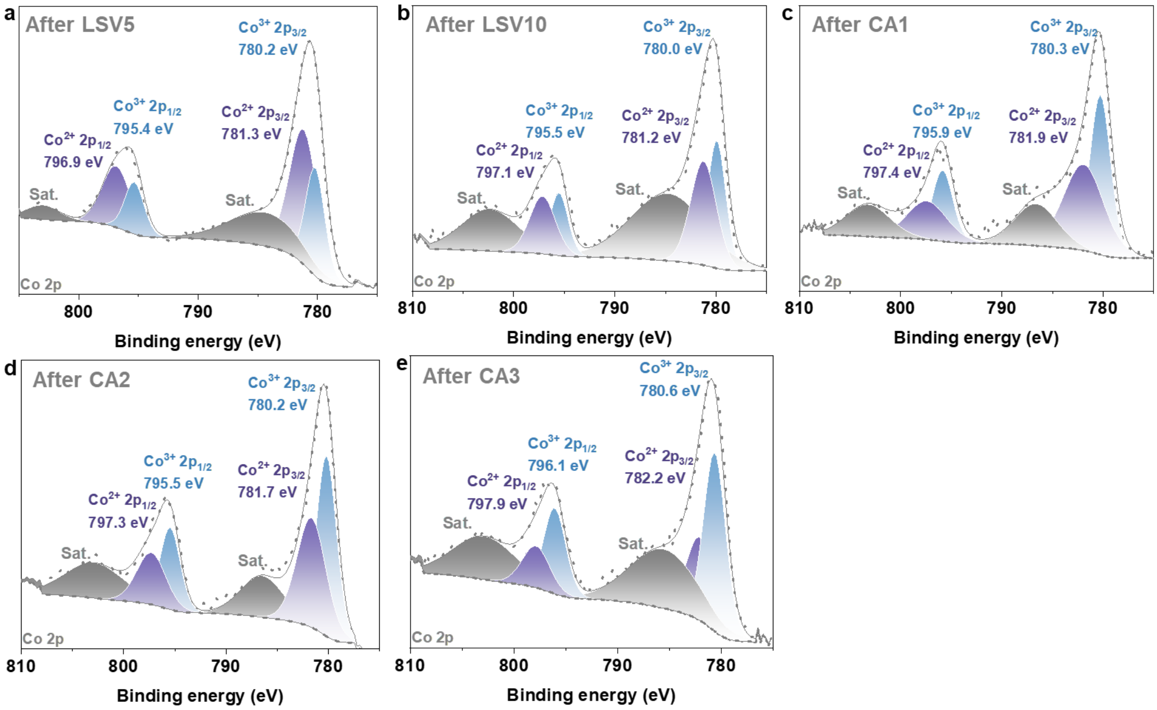

Fig. S32. XPS Co 2p spectra of **3** during studied OER process as indicated.

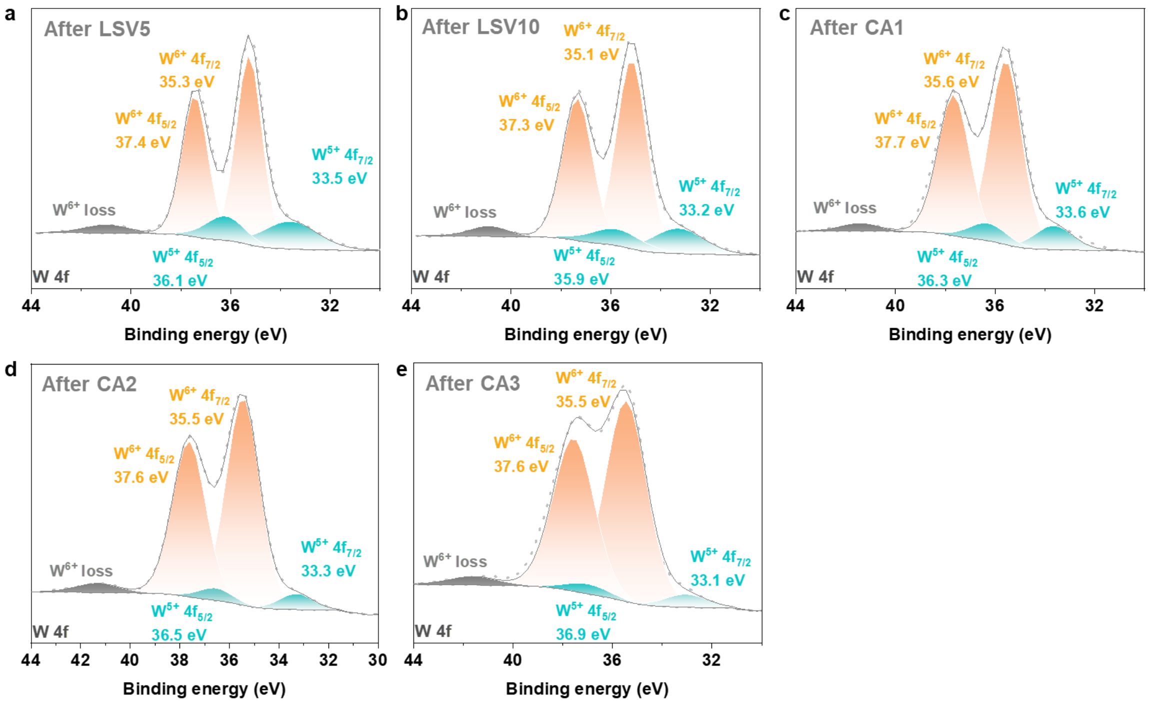

Fig. S33. XPS W 4f spectra of **3** during studied OER process as indicated.

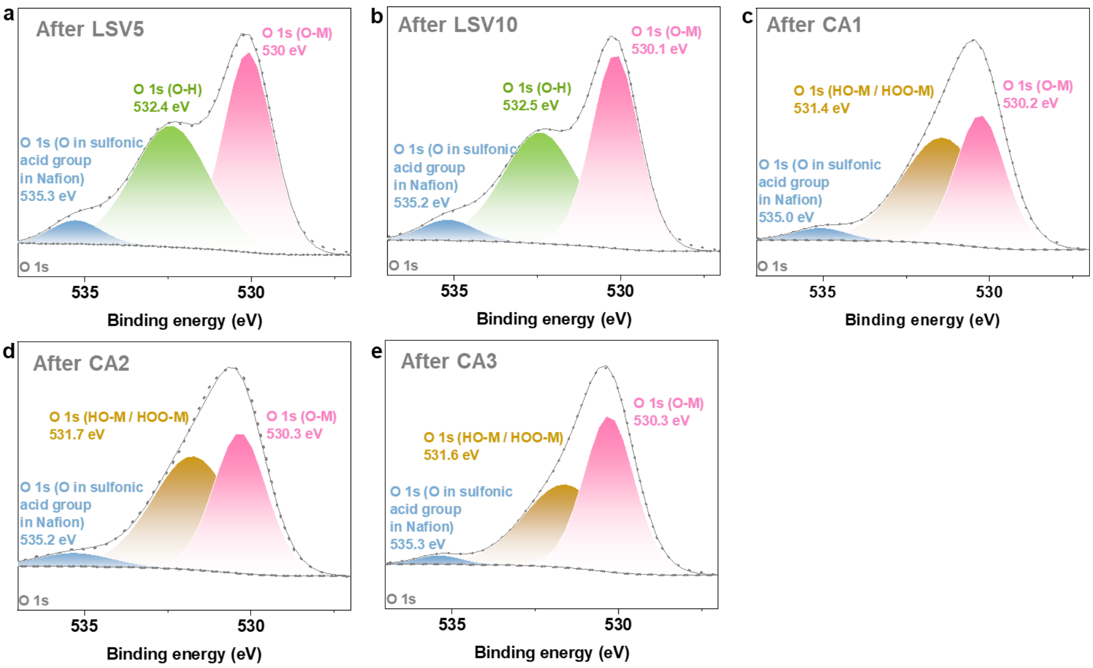

Fig. S34. XPS O 1s spectra of **3** during studied OER process as indicated.

Table S5. Dynamic surface transformation of **3** during studied OER process based on post-catalytic XPS analysis.

	Co^{2+} : Co^{3+}	W^{5+} : W^{6+}	HOO-M : O-M
Fresh	Co^{3+} free	1 : 1.3	HOO-M free
After LSV5	1 . 0.56	1 : 1.36	HOO-M free
After LSV10	1 : 0.83	1 : 1.45	HOO-M free
After CA1	1 : 1	1 : 1.65	0.76 : 1
After CA2	1 : 1.14	1 : 13	0.81 : 1
After CA3	1 : 1.72	1 : 15	1.15 : 1

6. Theoretical studies

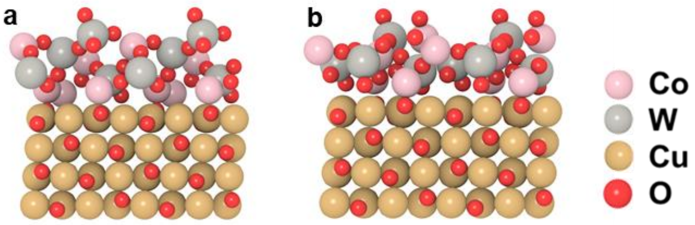

Fig. S35 Surface models for as-prepared **3** (a) and activated **3** after CA3 (b).

Table S6. The free energy correction from ΔZPE, $\Delta\int C_P\, dT$, $T\Delta S$, and the adsorption free energies of *OH, *O, *OOH intermediates on as-prepared **3** and activated **3** after CA3 surfaces.

Slab	Intermediates	ΔZPE (eV)	$\Delta\int C_P\, dT$ (eV)	$T\Delta S$ (eV)	ΔE_{ad} (eV)	ΔG_{ad} (eV)
As-prepared **3**	*OH	0.396	0.034	0.053	0.282	0.659
	*O (W site)	0.082	0.030	0.051	1.853	1.914
	*O (Co site)	0.068	0.036	0.069	2.483	2.518
	*OOH	0.463	0.076	0.143	3.473	3.869
Activated **3** after CA3	*OH	0.401	0.033	0.052	1.119	1.501
	*O (W site)	0.077	0.025	0.039	2.556	2.619
	*O (Co site)	0.077	0.030	0.054	2.419	2.472
	*OOH	0.440	0.057	0.101	3.748	4.144

Table S7. The free energies of the adsorption of OER intermediates, theoretical limiting potential (U_L) and overpotential (η) on as-prepared **3** and activated **3** after CA3 surfaces.

Slab	ΔG_1 (eV)	ΔG_2 (eV)	ΔG_3 (eV)	ΔG_4 (eV)	U_L (V)	η (V)
As-prepared **3**	0.66	1.26	1.95	1.05	1.95	0.72
Activated **3** after CA3	1.50	0.97	1.67	0.78	1.67	0.44

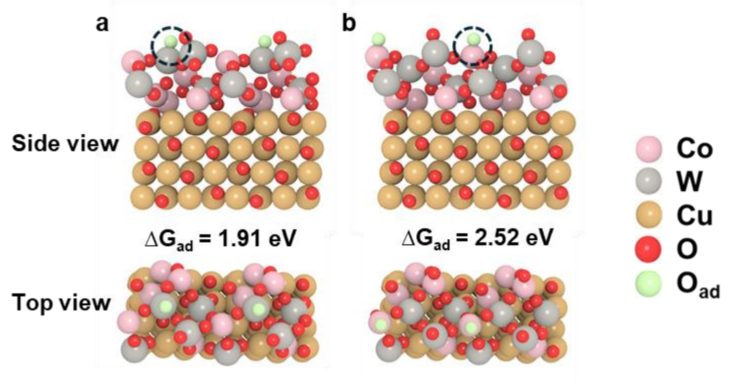

Fig. S36 Adsorption of O atom (O_{ad}) on the (a) W and (b) Co sites on the surface of as-prepared **3**.

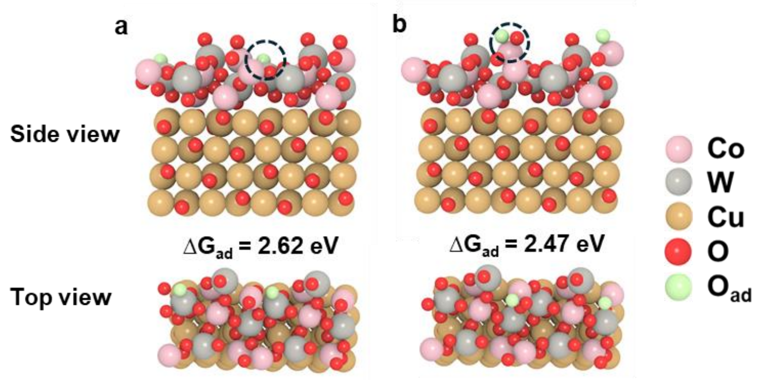

Fig. S37 Adsorption of O atom (O_{ad}) on the (a) W and (b) Co sites on the surface of activated **3** after CA3.

7. References:

[1] G. Kresse, D. Joubert, *Phys. Rev. B* **1999**, *59*, 1758–1775.

[2] G. Kresse, J. Furthmüller, *Comput. Mater. Sci.* **1996**, *6*, 15–50.

[3] J. P. Perdew, K. Burke, M. Ernzerhof, *Phys. Rev. Lett.* **1996**, *77*, 3865–3868.

[4] K. Mathew, R. Sundararaman, K. Letchworth-Weaver, T. A. Arias, R. G. Hennig, *J. Chem. Phys.* **2014**, *140*, 84106.

[5] K. Nishio, T. Miyazaki, H. Nakamura, *Phys. Rev. Lett.* **2013**, *111*, 155502.

[6] X. Wang, B. Liu, S. Ma, Y. Zhang, L. Wang, G. Zhu, W. Huang, S. Wang, *Nat. Commun.* **2024**, *15*, 2600.

[7] J. K. Nørskov, J. Rossmeisl, A. Logadottir, L. Lindqvist, J. R. Kitchin, T. Bligaard, H. Jónsson, *J. Phys. Chem. B* **2004**, *108*, 17886–17892.

[8] I. C. Man, H. Y. Su, F. Calle-Vallejo, H. A. Hansen, J. I. Martínez, N. G. Inoglu, J. Kitchin, T. F. Jaramillo, J. K. Nørskov, J. Rossmeisl, *ChemCatChem* **2011**, *3*, 1159–1165.

[9] L. Zhang, N. Zhang, H. Shang, Z. Sun, Z. Wei, J. Wang, Y. Lei, X. Wang, D. Wang, Y. Zhao, et al., *Nat. Commun.* **2024**, *15*, 9440.

[10] M. A. Hunter, J. M. T. A. Fischer, Q. Yuan, M. Hankel, D. J. Searles, *ACS Catal.* **2019**, *9*, 7660–7667.

[11] Q. Liang, G. Brocks, A. Bieberle-Hütter, *J. Phys. Energy* **2021**, *3*, 26001.

[12] V. Wang, N. Xu, J.-C. Liu, G. Tang, W.-T. Geng, *Comput. Phys. Commun.* **2021**, *267*, 108033.

[13] M. Yu, D. R. Trinkle, *J. Chem. Phys.* **2011**, *134*, 064111.

[14] S. Maintz, V. L. Deringer, A. L. Tchougréeff, R. Dronskowski, *J. Comput. Chem.* **2016**, *37*, 1030–1035.

[15] E. O. Oseghe, F. Guba, A. Misra, R. Gong, R. Liu, S. R. Waldvogel, D. Ziegenbalg, C. Streb, D. Gao, *Device* **2023**, *1*, 100020.

[16] A. Misra, I. Franco Castillo, D. P. Müller, C. González, S. Eyssautier-Chuine, A. Ziegler, J. M. de la Fuente, S. G. Mitchell, C. Streb, *Angew. Chem. Int. Ed.* **2018**, *57*, 14926–14931.

[17] D. Gao, S. Liu, R. Liu, C. Streb, *Chem. Eur. J.* **2020**, *26*, 11109–11112.

[18] J. Kibsgaard, T. F. Jaramillo, *Angew. Chem. Int. Ed.* **2014**, *53*, 14433–14437.

[19] R. Gong, D. Gao, R. Liu, D. Sorsche, J. Biskupek, U. Kaiser, S. Rau, C. Streb, *ACS Appl. Energy Mater.* **2021**, *4*, 12671–12676.

[20] L. N. Nguyen, U. T. D. Thuy, Q. D. Truong, I. Honma, Q. L. Nguyen, P. D. Tran, *Chem. Asian J.* **2018**, *13*, 1530–1534.

[21] S. M. Alshehri, J. Ahmed, T. Ahamad, P. Arunachalam, T. Ahmad, A. Khan, *RSC Adv.* **2017**, *7*, 45615–45623.

[22] M. Nakayama, A. Takeda, H. Maruyama, V. Kumbhar, O. Crosnier, *Electrochem. commun.* **2020**, *120*, 106834.

[23] D. Gao, R. Liu, J. Biskupek, U. Kaiser, Y.-F. Song, C. Streb, *Angew. Chem. Int. Ed.* **2019**, *58*, 4644–4648.

[24] D. Tantraviwat, S. Anuchai, K. Ounnunkad, S. Saipanya, N. Aroonyadet, G. Rujijanagul, B. Inceesungvorn, *J. Mater. Sci. Mater. Electron.* **2018**, *29*, 13103–13111.

[25] W. Luo, J. Hu, H. Diao, B. Schwarz, C. Streb, Y. F. Song, *Angew. Chem. Int. Ed.* **2017**, *56*, 4941–4944.

7. Author contributions

C.N., R.L. and D.G. conceived the idea for the project. C.N. and D.L.T. carried out the material synthesis. C.N., Z.D., M.O.C., D.A., K.S., B.M., L.P., B.F.M. contributed to material characterization. C.N. and D.L.T. designed and performed the electrocatalytic studies. X.L. and M.H. performed DFT calculation. R.L., X.L. and D.G. acquired funding and provided project administration. The manuscript was written and reviewed through contributions of all authors. All authors have given approval to the final version.
